# Targeting the γ-/β-secretase interaction reduces β-amyloid generation and ameliorates Alzheimer’s disease-related pathogenesis

**DOI:** 10.1038/celldisc.2015.21

**Published:** 2015-08-18

**Authors:** Jin Cui, Xiaoyin Wang, Xiaohang Li, Xin Wang, Chenlu Zhang, Wei Li, Yangming Zhang, Haifeng Gu, Xin Xie, Fajun Nan, Jian Zhao, Gang Pei

**Affiliations:** 1 State Key Laboratory of Cell Biology, Institute of Biochemistry and Cell Biology, Shanghai Institutes for Biological Sciences, Chinese Academy of Sciences; Graduate School, University of Chinese Academy of Sciences, Chinese Academy of Sciences, Shanghai, China; 2 State Key Laboratory of Drug Research, The National Center for Drug Screening, Shanghai Institute of Materia Medica, Chinese Academy of Sciences, Shanghai, China; 3 School of Life Science and Technology, Collaborative Innovation Center for Brain Science, Tongji University, Shanghai, China; 4 Translational Medical Center for Stem Cell Therapy, Shanghai East Hospital, School of Medicine, Tongji University, Shanghai, China

**Keywords:** PS1/BACE1 interaction, Aβ generation, Alzheimer’s disease

## Abstract

Despite decades of intense global effort, no disease-modifying drugs for Alzheimer’s disease have emerged. Molecules targeting catalytic activities of γ-secretase or β-site APP-cleaving enzyme 1 (BACE1) have been beset by undesired side effects. We hypothesized that blocking the interaction between BACE1 and γ-secretase subunit presenilin-1 (PS1) might offer an alternative strategy to selectively suppress Aβ generation. Through high-throughput screening, we discovered that 3-α-akebonoic acid (3AA) interferes with PS1/BACE1 interaction and reduces Aβ production. Structural analogs of 3AA were systematically synthesized and the functional analog XYT472B was identified. Photo-activated crosslinking and biochemical competition assays showed that 3AA and XYT472B bind to PS1, interfere with PS1/BACE1 interaction, and reduce Aβ production, whereas sparing secretase activities. Furthermore, treatment of APP/PS1 mice with XYT472B alleviated cognitive dysfunction and Aβ-related pathology. Together, our results indicate that chemical interference of PS1/BACE1 interaction is a promising strategy for Alzheimer’s disease therapeutics.

Alzheimer’s disease (AD) is a progressive neurodegenerative disorder, and is the most common form of dementia worldwide, causing progressive memory loss and severe cognitive dysfunction [1]. Amyloid plaques formed by amyloid-β peptides (Aβ) have been identified as one of the major pathological hallmarks of AD [2]. Mounting evidence suggests that reduction of brain Aβ either by directly reducing its production and aggregation, or by promoting its degradation and clearance, could ameliorate AD pathology [3–5]. The Aβ peptides are formed after sequential cleavage of the Aβ precursor protein (APP) catalyzed by β-site APP-cleaving enzyme 1 (BACE1) and γ-secretase [6]. BACE1, a type I transmembrane aspartic acid protease, initiates amyloidogenic APP processing [7]. Recent systematic proteomic studies have identified an extensive list of substrates that can potentially be cleaved by BACE1 [8]. A number of BACE1 substrates have been reported to have vital roles in neuronal functions [9, 10]. γ-secretase is a plasma membrane-embedded complex, consisting of at least four proteins: presenilin-1 (PS1), nicastrin (NCT), anterior pharynx-defective phenotype 1 and presenilin enhancer 2. PS1, an aspartyl protease, is the catalytic subunit of the complex. γ-secretase complex mediates the intramembrane proteolysis of APP and other transmembrane proteins, including Notch and cadherins. Cleavage of these proteins by γ-secretase releases active cytoplasmic peptide fragments that possess important biological functions [11–14].

The primary approaches to reduce Aβ generation have focused on the direct inhibition or modulation of the protease activities of BACE1 or γ-secretase [4, 15]. Typical inhibitors bind to the catalytic domains of these secretases thus blocking their proteolytic activities [16, 17]. However, because BACE1 and γ-secretase are versatile proteases with as many as hundreds of substrates, modulation of BACE1 or γ-secretase activity may pose the risk of interfering with critical signaling processes [14, 18]. Recently γ-secretase modulators (GSMs) have been developed to target specifically the γ-site cleavage of APP and reduce Aβ42 levels without altering overall γ-secretase activity [19]. In addition, recent studies and our previous studies have shown that some secretase-binding proteins can modulate substrate specificity by altering the conformation or subcellular localization of the secretases [20–23]. These findings provide promising alternatives for specifically modulating amyloidogenic processing of APP by BACE1 or γ-secretase.

Despite a growing list of BACE1 and γ-secretase substrates, limited proteins are processed by both BACE1 and γ-secretase [24], and APP is a notable exception. Previous studies have reported that PS1 interacts with BACE1 and regulates its expression and activity [25, 26]. Furthermore, using a flotation sucrose density gradient centrifugation assay a fraction of BACE1 has been detected on the Flotillin-2-positive lipid rafts, where a large population of γ-secretase also reside [27]. However, little research has been carried out to explore the consequences of disrupting the interaction between BACE1 and γ-secretase. We hypothesized that PS1/BACE1 interaction might contribute to the efficiency of amyloidogenic proteolysis of APP and asked if it is possible, by targeting the PS1/BACE1 interaction, we could reduce Aβ generation without affecting BACE1 or γ-secretase activities. Hence, we performed interaction-based high-throughput screen for PS1/BACE1 interaction-blocking compounds. We found 3-α-Akebonoic acid and its structural analog XYT472B with the ability to interfere with PS1/BACE1 interaction by binding to PS1 and to reduce Aβ generation without affecting BACE1 or γ-secretase activities. Furthermore, the cognitive impairment as well as the Aβ-related pathology of APP/PS1 transgenic mice could be ameliorated by XYT472B treatment.

## Results

### Monitoring the PS1/BACE1 interaction with FRET and Split-TEV assay

**Figure 1:**
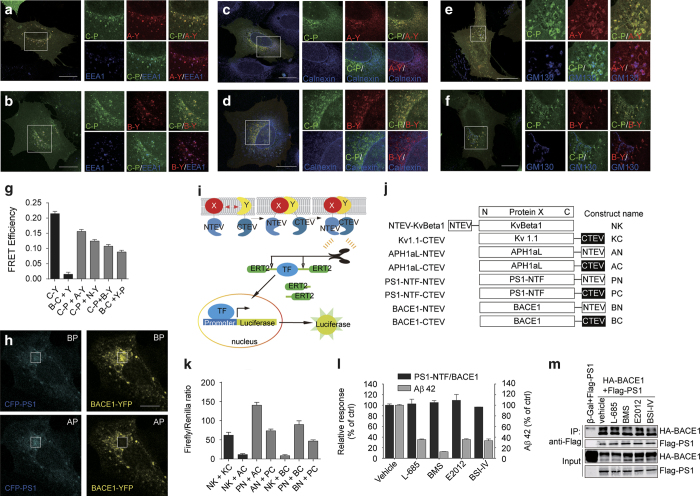
Monitoring the cellular PS1/BACE1 interaction in FRET assay and Split-TEV assay. (**a**–**f**) Subcellular co-localization of CFP-PS1 (C-P) and APH1aL-YFP (A-Y), CFP-PS1 and BACE1-YFP (B-Y) with EEA1, calnexin and GM130 in the presence of the other γ-secretase subunits in U-2 OS cells. Forty-eight hours after transfection, cells overexpressing CFP-PS1, APH1aL-YFP, NCT and BACE1-YFP were fixed and counter-stained for EEA1, calnexin and GM130. Scale bar, 20 μm. White box indicates the enlarged area. (**g**) Acceptor photobleaching FRET analysis in HEK293 cells. A CFP-YFP (C-Y) fusion protein was used as a positive control, and BACE1-CFP (B-C)/YFP (Y) pair as a negative control. The intermolecular FRET efficiency of PS1 and BACE1, assayed using two fluorescent protein pairs: CFP-PS1 (C-P) and BACE1-YFP (B-Y), B-C and YFP-PS1 (Y-P), is lower than that of C-P/APH1aL-YFP (A-Y) and comparable with that of C-P/NCT-YFP (N-Y). *n*=20 cells per group. (**h**) Representative image of CFP-PS1/BACE1-YFP FRET in the presence of other γ-secretase components in HEK293 cells. Scale bar, 10 μm. White box indicates the photobleached area. (**i**) A schematic of the reporter-based split-TEV assay for monitoring protein–protein interaction and (**j**) the fusion-protein constructs used in this study. (**k**) Interaction of PS1 and BACE1 in HEK293MSR cells monitored by luciferase-based split-TEV assay. Luciferase reporter activities were measured 24 h after transfection. NTEV-KvBeta1 (NK)+Kv1.1-CTEV (KC) heterodimers and PS1-NTF-NTEV (PN)+APH1aL-CTEV (AC) were positive controls, NK+AC and NK+BACE1-CTEV (BC) were negative controls. PN+BC and BACE1-NTEV (BN)+PS1-NTF-CTEV (PC) showed high interaction signals, indicating an interaction between PS1-NTF and BACE1. (**l**) PS1-NTF/BACE1 split-TEV reporter activities and Aβ42 production in cells treated for 16 h with 1 μM chemicals indicated in the *x*-axis . (**m**) Co-immunoprecipitation of C-terminal Flag-tagged PS1 (Flag-PS1) and C-terminal HA-tagged BACE1 (HA-BACE1). HEK293T cells overexpressing HA-BACE1 and β-Gal or Flag-PS1 were treated with 3 μM chemicals for 16 h before cell lysis and immunoprecipitation. AP, after photobleaching; BP, before photobleaching.

We examined the subcellular localization of BACE1 and PS1 in U-2 OS cells by immunofluorescence microscopy using CFP-PS1, APH1aL-YFP or BACE1-YFP, and compared with the localization of three secretory pathway markers: calnexin for the endoplasmic reticulum, GM130 for the Golgi complex and EEA1 for endosome. As shown in Figure 1a, CFP-PS1 exhibited punctate pattern. The CFP-PS1 puncta co-localized with those of anterior pharynx-defective phenotype 1aL, as well as EEA1, calnexin and GM130, indicating that CFP-PS1 recapitulates the cellular localization of γ-secretase (Figures 1a, c and e). BACE1-YFP co-localized with EEA1 (Figure 1b), but not with calnexin (Figure 1d) and only slightly with GM130 (Figure 1f), suggesting BACE1-YFP recapitulates largely the subcellular distribution of endogenous BACE1 [28]. Importantly, the overlapping distribution of CFP-PS1 and BACE1-YFP in early endosomes and the Golgi apparatus, suggests a potential and functional interaction between BACE1 and PS1 in cells. This is consistent with earlier studies reporting a close association between BACE1 and PS1 [28–30].

To explore the interaction between BACE1 and PS1 in greater details, we adopted FRET and split-TEV assays. Fusion protein CFP-YFP was used as a robust positive control while BACE1-CFP and YFP pair served as negative controls. As shown in Figures 1g and h, FRET efficiency in both CFP-PS1/BACE1-YFP and BACE1-CFP/YFP-PS1 pairs was ~0.1, a value comparable to the FRET efficiency of the interaction between γ-secretase subunits CFP-PS1/APH1aL-YFP and CFP-PS1/NCT-YFP. These results suggest a close proximity of PS1 and BACE1.

In parallel, we designed a split-TEV assay to quantitatively monitor the interaction of PS1 with BACE1. The split-TEV assay is based on bimolecular enzymatic activity complementation of TEV protease (Figure 1i) [31], which is well-suited to high-throughput screening. Heterodimers of potassium channel NTEV-KvBeta1 and Kv1.1-CTEV were used as a positive control, whereas NTEV-KvBeta1/APH1aL-CTEV and NTEV-KvBeta1/BACE1-CTEV were used as negative controls (Figure 1k). The interaction between γ-secretase subunits PS1-NTF (N-terminal fragment) and APH1aL led to an increase in luciferase reporter activities comparable to that of NTEV-KvBeta1 and Kv1.1-CTEV, validating our assay. We found that luciferase reporter activities of cells expressing PS1-NTF-NTEV/BACE1-CTEV and cells expressing PS1-NTF-CTEV/BACE-NTEV were comparable to that of cells carrying APH1aL-NTEV/PS1-NTF-CTEV. These results confirm the interaction between PS1 and BACE1.

We then asked whether typical secretase inhibitors or modulators could affect the interaction between PS1 and BACE1. We tested γ-secretase inhibitors (GSIs) L-685 458 and BMS-708163, GSM E2012, and BACE1 inhibitor-IV. At their respective IC_90_ concentration, these chemicals reduced Aβ generation significantly, but had little effect on PS1/BACE1 interaction as monitored by split-TEV luciferase reporter assay (Figure 1l) or by co-immunoprecipitation assay (Figure 1m). These results indicate that inhibition or modulation of γ-secretase or BACE1 does not interfere with PS1/BACE1 interaction.

### Interaction-based screen identifies small molecule BBP18-H10 that interferes with PS1/BACE1 interaction and reduces Aβ generation

**Figure 2:**
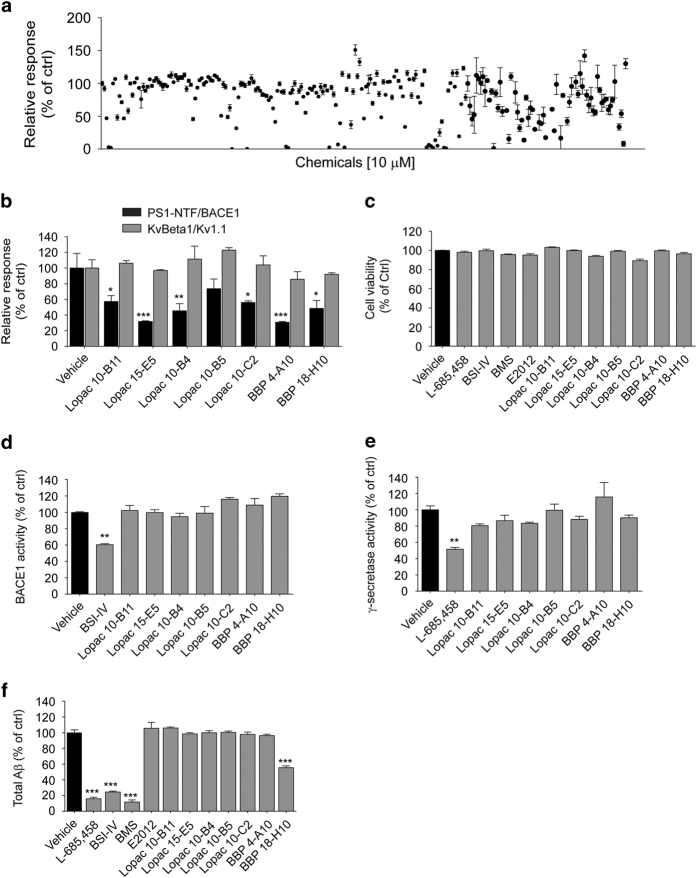
Screening of PS1/BACE1 interaction-interfering chemicals. (**a**) Representative results of the screen for chemicals that interfere with PS1-NTF-TEV/BACE1-CTEV interaction. Two hours after transfection, HEK293MSR cells were treated with 10 μM chemicals for 16 h and the luciferase reporter activities were measured. (**b**) PS1-NTF/BACE1 and KvBeta1/Kv1.1 split-TEV reporter activities. Following transfection, cells were treated for 16 h with 10 μM candidate compounds. (**c**) Cell viability of HEK293/APPswe cells. HEK293/APPswe were treated with 1 μM L-685 458, BACE1 inhibitor-IV (BSI-IV), BMS-708163, E2012 or 10 μM candidate compounds for 8 h and subjected to cell viability test. (**d**) BACE1 and (**e**) γ-secretase activity in fluorogenic substrate assay. HEK293T cell membrane fractions were incubated with 10 μM chemicals. (**f**) Total Aβ production of HEK293/APPswe cells. Cells were treated with 1 μM L-685 458, BSI-IV or BMS, or 10 μM candidate chemicals for 8 h. **P<0.01 determined by one-way ANOVA.

We established a PS1-NTF-NTEV/BACE1-CTEV split-TEV assay-based high-throughput screening system and employed it to search multiple chemical libraries of over 10 000 small molecules (representative screening results are shown in Figure 2a, Supplementary Table S1). Seven small molecules were identified that reduced the PS1-NTF-NTEV/BACE1-CTEV interaction signal specifically, with little effect on NTEV-KvBeta1/Kv1.1-CTEV interaction (Figure 2b), cell viability (Figure 2c), or secretase activities (Figures 2d and e). We further tested the effects of these molecules on Aβ production using HEK293/APP Swedish mutant (APPswe) cells. Upon an 8 h treatment, only BBP18-H10 reduced Aβ production significantly (Figure 2f). We thus focused on BBP18-H10 for further study.

### 3-α-Akebonoic acid and its structural analog XYT472B interfere with PS1/BACE1 interaction and reduce cellular Aβ generation

**Figure 3:**
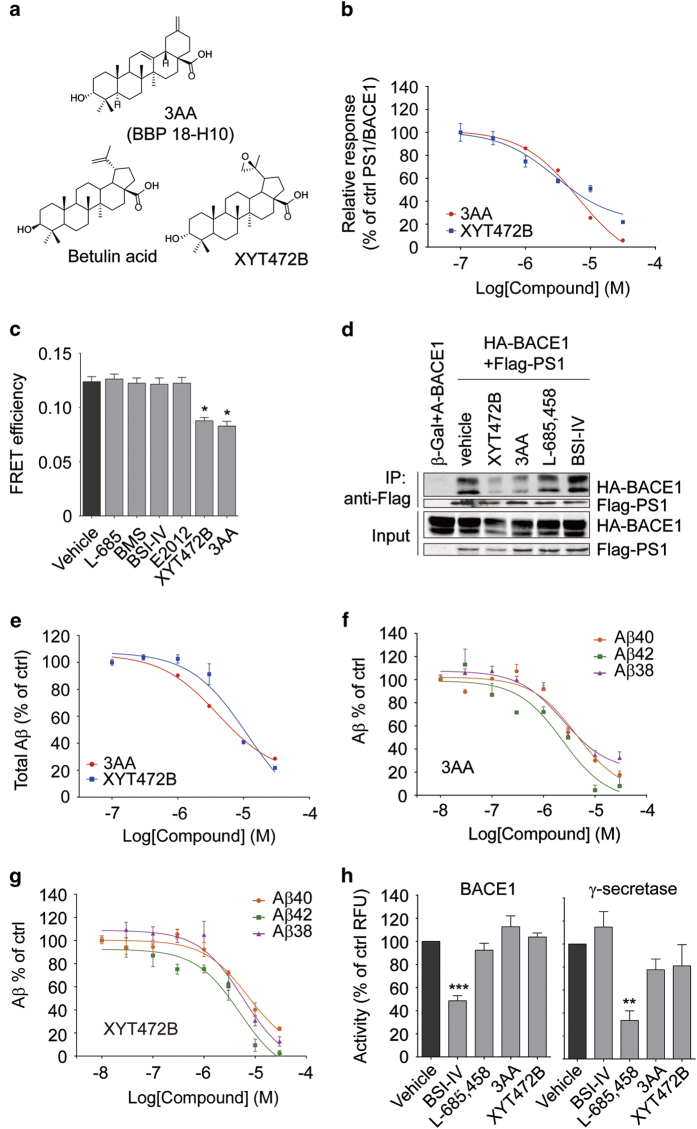
3AA and its structural analog XYT472B reduce PS1/BACE1 interaction and Aβ production. (**a**) Chemical structures of 3-α-Akebonoic acid (3AA, BBP 18-H10), betulin acid and XYT472B. (**b**) 3AA and XYT472B reduce PS1-NTF/BACE1 interaction dose-dependently in split-TEV assay. Cells were treated with different concentrations of 3AA or XYT472B for 16 h. (**c**) 3AA and XYT472B reduce FRET efficiency of PS1 and BACE1. Cells (overexpressing fusion protein CFP-PS1, BACE1-YFP, APH1aL, NCT and Pen2) were treated with 3 μM chemicals for 16 h and subjected to acceptor photobleaching FRET analysis. (**d**) XYT472B and 3AA reduce PS1/BACE1 interaction in co-immunoprecipitation assay. HEK293T cells overexpressing C-terminal HA-tagged BACE1 and β-Gal or C-terminal Flag-tagged PS1 were treated with 3 μM chemicals for 16 h before cell lysis and immunoprecipitation. (**e**) 3AA and XYT472B reduce total Aβ production. HEK293/APPswe cells were treated with 3AA or XYT472B for 8 h and the culture media were collected for sandwich ELISA to quantify the total Aβ production. (**f**) 3AA and (**g**) XYT472B dose-dependently decrease Aβ40, Aβ42 and Aβ38 generation. HEK293/APPswe cells were treated with different concentrations of chemicals for 8 h before the supernatants were collected for ELISA quantification. (**h**) 3AA and XYT472B show no significant effects on BACE1 activity (left) or γ-secretase activity (right). HEK293T cell membrane fractions with 10 μM chemicals were incubated with fluorogenic substrates and the fluorescent signal from processed substrates were monitored and presented. *P<0.05, **P<0.01, ***P<0.001, determined by one-way ANOVA.

BBP18-H10 is 3-α-Akebonoic acid (3AA, Figure 3a), a natural compound from *Akebia quinata*. Purification of 3AA from *A quinata* is complicated and difficult [32], and the amount of 3AA that we could obtain was limited, hindering further investigation. To obtain more selective and potent compounds that could interfere with PS1/BACE1 interaction, we constructed a focused natural product library of betulin acid derivatives, based on betulin acid’s structural similarity to the hit compound 3AA. Modifications focused on three structural moieties (C-3 hydroxyl group, C-17 carboxyl group and C-20 double bond) of betulin acid, and we found that the 3-α-hydroxyl group was essential for the inhibitory activity. Replacement of C-20 double bond with epoxide group led to an increase of inhibitory activity and decrease of the toxicity, whereas modifications at C-17 carboxyl group were unsuccessful (data not shown). Finally XYT472B (Figure 3a) was identified as the most potent compound. As shown in Figure 3b, XYT472B displayed similar inhibitory potency as 3AA on PS1-NTF/BACE1 interaction when monitored in split-TEV assay. Interference with the PS1/BACE1 interaction by either 3AA or XYT472B was further confirmed by FRET analysis and co-immunoprecipitation assays, whereas none of the GSIs or BACE inhibitors interfered with the interaction of PS1 and BACE1 as monitored by FRET (Figure 3c) and co-immunoprecipitation assay (Figure 3d).

We next investigated cellular Aβ production following 3AA or XYT472B treatment. Both 3AA and XYT472B reduced total Aβ production in HEK293/APPswe cells in a dose-dependent manner (Figure 3e). 3AA (Figure 3f) and XYT472B (Figure 3g) also reduced the production of Aβ40, Aβ42 and Aβ38 in a similar kinetics in HEK293/APPswe cells, with an IC_50_ of 3 μM. We next explored the effects of 3AA and XYT472B on secretase activity in an *in vitro* assay. Whereas BACE1 inhibitor-IV significantly inhibited BACE1 activity, GSI L-685 458, 3AA and XYT472B showed little effect (Figure 3h, left). Conversely, L-685 458 significantly inhibited processing of a fluorogenic substrate by γ-secretase, but BACE1 inhibitor-IV, 3AA and XYT472B did not (Figure 3h, right). These data indicate that 3AA and its analog XYT472B reduce Aβ production without directly inhibiting BACE1’s and γ-secretase’s enzymatic activities.

**Figure 4:**
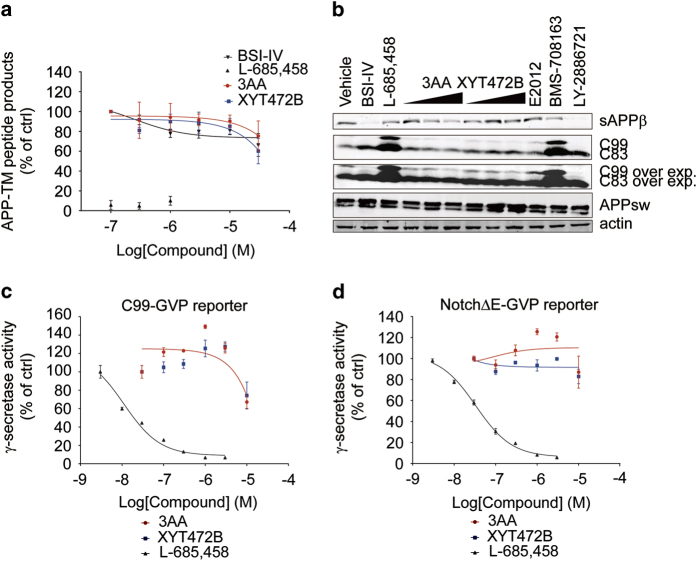
3AA and XYT472B do not inhibit secretase activities. (**a**) ELISA-based γ-secretase activity assay. HEK293T membrane fractions were incubated with chemicals and APP-TM peptides for 2 h before the ELISA analysis of the processed products. (**b**) Cellular APP processing was unaffected by 3AA or XYT472B treatment. HEK293/APPswe cells were incubated with chemicals for 4 h. Cell lysates were prepared and subjected to western blotting analysis of C99, C83 and APPswe. Culture media were collected for sAPPβ analysis. (**c** and **d**) Cellular γ-secretase activity was monitored and found unaltered in (**c**) C99-GVP and (**d**) NotchΔE-GVP reporter assays. Two hours after transfection, HEK293T cells were treated with chemicals. Luciferase reporter activities were measured 24 h after transfection.

We further investigated if 3AA or XYT472B could interfere with the cleavage of APP by γ-secretase or BACE1. First, we used a biotinylated peptide that contains the transmembrane domain of APP (APP-TM) to monitor γ-secretase activity [33]. Membrane fractions extracted from HEK293T cells were incubated with APP-TM peptide and different concentrations of compounds. Processed biotinylated p-40 peptides were captured by streptavidin-coated 96-well plates and detected by an anti-amyloid-β40 antibody (clone G2–10). As expected, 100 nM L-685 458 inhibited p-40 peptide production almost completely but BACE1 inhibitor-IV showed no effect (Figure 4a), and neither 3AA nor XYT472B at 10 μM inhibited p-40 peptide production. We also assessed the cleavage pattern of APP in the presence of either 3AA or XYT472B (Figure 4b) on western blots. Treatment with BACE1 inhibitor-IV and another BACE1 inhibitor, LY-2886721, abolished secreted fragment sAPPβ and C99 production completely, and caused a slight accumulation of intracellular fragment C83, whereas treatments of GSI L-685 458 and BMS-708163 caused significant accumulation of C99 and another intracellular fragment C83, and GSM E2012 showed little effect on any of the APP processing products. In contrast to BACE1 inhibitor-IV and GSIs, but similar to E2012, neither 3AA nor XYT472B treatment led to significant changes in the production of sAPPβ or intracellular C99/C83. Consistent results were also obtained in C99-GVP and NotchΔE-GVP reporter assays. As shown in Figure 4c, L-685 458 had an inhibitory IC_50_ of <100 nM on both C99-GVP and NotchΔE-GVP reporter activities, whereas 3AA and XYT472B only moderately inhibited proteolytic processing of C99-GVP and NotchΔE-GVP (Figure 4d).

### Photoclickable analog XYT1032 reveals binding sites of XYT472B and 3AA on PS1

**Figure 5:**
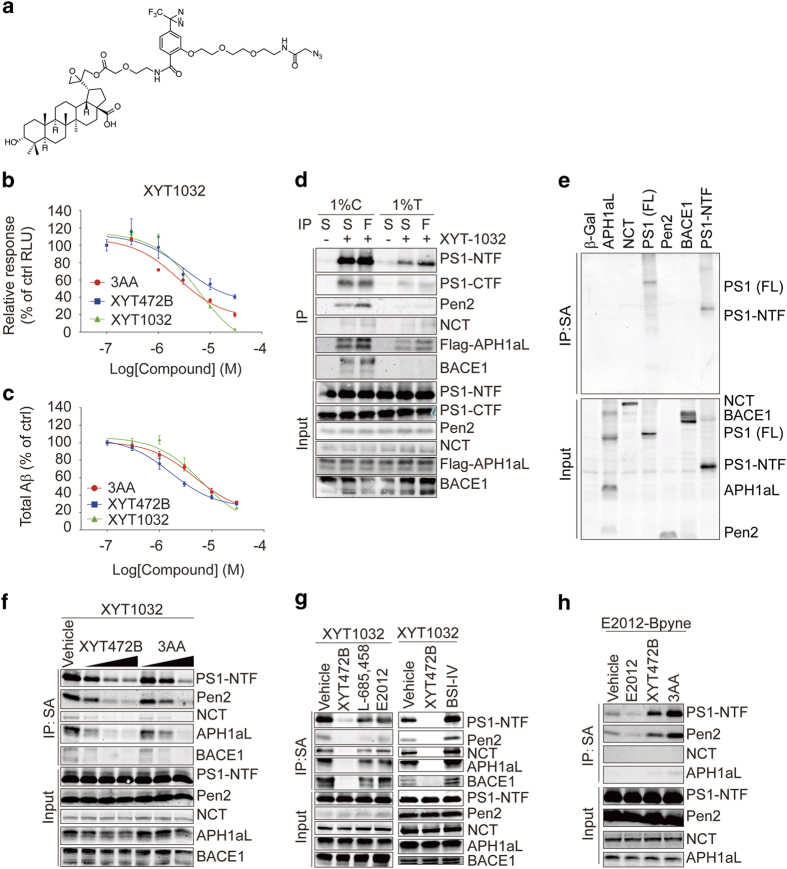
Photoclickable analog XYT1032 reveals XYT472B and 3AA binding sites on PS1. (**a**) Chemical structure of XYT1032. (**b** and **c**) XYT1032 reduces PS1-NTF/BACE1 interaction in split-TEV assay (**b**) and total Aβ production (**c**). Transfected HEK293MSR cells were treated with chemicals for 16 h before DLR analysis of luciferase reporter activities, and HEK293/APPswe cells were treated for 8 h before the supernatants were collected for ELISA. (**d**) Labeling and affinity purification of γ-secretase subunits and BACE1 by XYT1032 in different detergents. Cells expressing PS1, Pen2, NCT, C-terminal Flag-tagged APH1aL and BACE1 were treated with XYT1032 (3 μM) and photo-activated click reaction were carried out. γ-secretase subunits and BACE1 were affinity purified by anti-Flag or straptavidin resin as indicated. (**e**) XYT1032 binds only to PS1. Cells expressing β-Gal, C-terminal Flag-tagged APH1aL, NCT, PS1 (FL), Pen2, BACE1 or PS1-NTF, respectively, were treated with XYT1032 (3 μM) and subjected to photo-activated click reaction and affinity purification. (**f**) XYT472B and 3AA compete with XYT1032 in labeling of PS1-NTF. Cells were treated with 3 μM XYT1032 in the absence or presence of 10 μM, 30 μM, 100 μM XYT472B or 3AA for 2 h. (**g**) Secretase inhibitors and modulator fail to compete with XYT1032 in binding to PS1-NTF. Cells were incubated with 3 μM XYT1032 in the absence or presence of 100 μM designated chemicals. (**h**) XYT472B and 3AA enhanced the labeling of PS1-NTF by E2012-BPyne. Cells were incubated with 1 μM E2012-BPyne in the absence or presence of 50 μM designated chemicals for 1 h. After chemical incubation, photo-activated crosslinking, click reaction and streptavidin affinity purification in 1% CHAPSO buffer were performed. C, CHAPSO; F, Flag; S, streptavidin; T, TritonX-100.

Photo-affinity labeling by activity-based probes can be applied in identifying the target of small molecules [34]. On the basis of structure-activity analysis of XYT472B and its congeners, we designed and synthesized an activity-based probe XYT1032 (Figure 5a), which displayed an inhibitory potency comparable to that of XYT472B and 3AA in both PS1-NTF/BACE1 split-TEV assay and cellular Aβ production experiment (Figures 5b and c), suggesting XYT1032 can be used to directly label its targets in live cells.

First, we tested whether XYT1032 binds to BACE1 and/or γ-secretase. We prepared 1% CHAPSO soluble and 1% Triton X-100 soluble cell lysates from HEK293T cells expressing BACE1 and four γ-secretase subunits. After photo-click reactions, XYT1032-labeled proteins were enriched by streptavidin slurry and compared with proteins immunopurified using anti-FLAG slurry. A considerable amount of BACE1 formed complexes with γ-secretase in 1% CHAPSO extracts and was pulled down by immunoprecipitation of C-terminal Flag-tagged APH1aL (Figure 5d, lane 3). In 1% Triton X-100 extracts, the interaction between γ-secretase subunits and BACE1 was disrupted (Figure 5d, lane 6). We found that BACE1 and all γ-secretase subunits were detected in XYT1032-labeled proteins purified from 1% CHAPSO extracts (Figure 5d, lane 2), whereas in XYT1032-labeled 1% Triton X-100 extracts only PS1, with small amounts of NCT, APH1aL and Pen2 were detectable (Figure 5d, lane 5), indicating that XYT1032 binds to γ-secretase but not BACE1. To further identify which γ-secretase subunit XYT1032 binds to, we expressed the secretase subunits separately in HEK293T cells. XYT1032-labeled proteins were purified with streptavidin resin under more stringent condition (1% Triton X-100, 0.3% SDS). Interestingly, only PS1, especially the NTF of PS1, was enriched by XYT1032 labeling and streptavidin affinity purification (Figure 5e, lane 4 and 7). Taken together, these results indicate that XYT1032 binds to γ-secretase subunit PS1.

We tested whether the covalent conjugation of XYT1032 to PS1 resembles the binding properties of XYT472B and 3AA. For this, we used XYT472B and 3AA to compete with XYT1032 in the labeling of PS1-NTF. As shown in Figure 5f, in the presence of XYT472B or 3AA, the labeling of PS1-NTF was decreased dose dependently, suggesting that XYT472B and 3AA bind to PS1 directly and share similar binding sites with XYT1032. Furthermore, we confirmed that 3AA and XYT472B modulates C99 processing by γ-secretase but not BACE1 using an *in vitro* C99 assay in which BACE1 was knocked down by ~50% (Supplementary Figure S1a), but the inhibitory effects of 3AA and XYT472B on APP intracellular domain (AICD) production remained unchanged (Supplementary Figure S1b).

We then asked whether the binding site(s) of XYT1032 are similar to or different from those of typical secretase inhibitors or modulators by using several GSIs and GSMs, as well as BACE1 inhibitor-IV to compete with XYT1032 for the labeling of PS1-NTF. L-685 458 is a conventional γ-secretase inhibitor that targets the γ-secretase catalytic site [17], whereas E2012, an imidazole class GSM, binds to PS1-NTF at a different site from L-685 458 [35], and BACE1 inhibitor-IV binds to the catalytic pockets of BACE1 [36]. As shown in Figure 5g, labeling of XYT1032 to PS1-NTF was significantly attenuated in the presence of XYT472B whereas L-685 458, E2012 and BACE1 inhibitor-IV showed little effect. A reversed competition photo-click labeling experiment was performed using the E2012-derived probe E2012-BPyne [35]. As shown in Figure 5h, the labeling of E2012-BPyne to PS1, as well as APH1aL and Pen2, was significantly enhanced in the presence of either XYT472B or 3AA, suggesting that the binding of 3AA and its analogs to PS1 is distinct from both L-685 458 and E2012 and might allosterically influence the binding of E2012. Taken together, these results indicate that XYT1032 does not bind to BACE1 but binds to PS1, and it binds to different sites on PS1 from L-685 458 and E2012.

### 3AA and its analogs bind to the sixth transmembrane domain of PS1 without affecting γ-secretase activity

**Figure 6:**
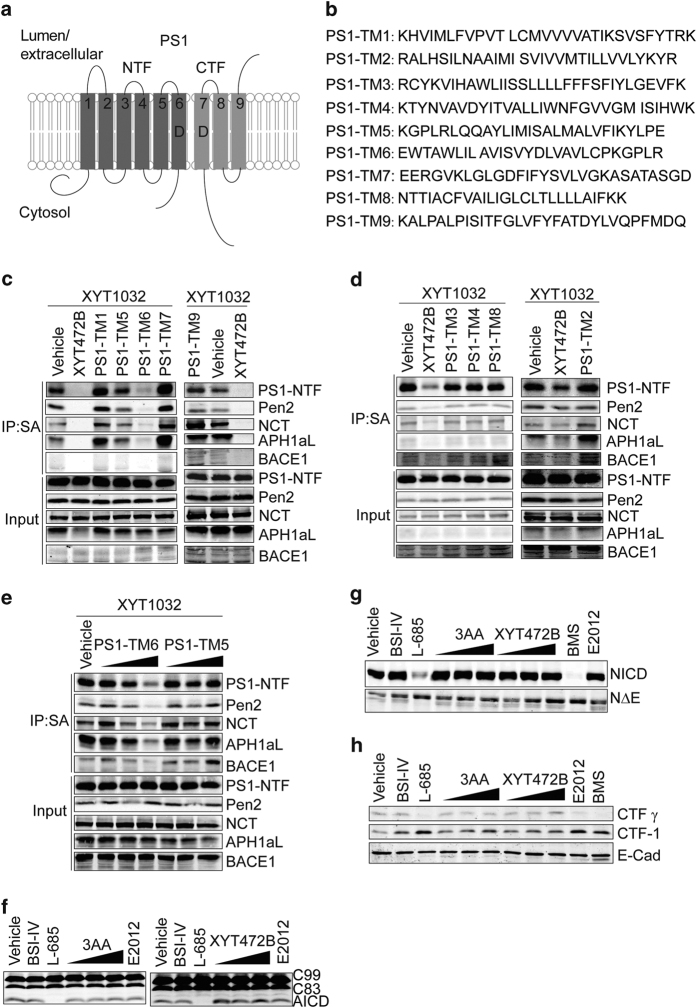
3AA and its analogs bind to the sixth transmembrane domain of PS1 without affecting γ-secretase activity. (**a**) A schematic of PS1 transmembrane domain-derived peptides. (**b**) Amino acid sequences of PS1-TM peptides. (**c** and **d**) Competition experiments of PS1 TM peptides to the photo-affinity labeling of PS1-NTF, other γ-secretase components and BACE1. Vehicle (DMSO), 50 μM XYT472B or PS1-TM peptides were pre-incubated in medium with XYT1032 for 15 min prior to the 2 h cellular treatment of transfected HEK293T cells. (**e**) PS1-TM6 peptide competes with PS1-NTF in the labeling of XYT1032. PS1-TM6 and PS1-TM5 peptide (10 μM, 20 μM and 50 μM) were pre-incubated with XYT1032 for 15 min in medium, and then added to the cells. After treatment, cells were lysed with 1% CHAPSO buffer and subjected to photo-activated crosslinking, click reaction and streptavidin purification. (**f**) 3AA and XYT472B exhibited little effect on AICD production in *in vitro* C99 assay. HEK293T cell membrane fractions were incubated with chemicals for 4 h. (**g**) Processing of NotchΔE in HEK293T cells was unaffected by 3AA- or XYT472B treatment. (**h**) 3AA and XYT472B do not affect E-Cadherin processing *in vitro*. The concentration of BSI-IV, L-685 458, E2012, BMS used in (**f** and **h**) was 10 μM.

To further map the possible binding regions of XYT1032 on PS1, we designed nine peptides based on the predicted PS1 transmembrane domains [12] (PS1-TM1~9, Figures 6a and b). Photo-click affinity labeling experiments with XYT1032 were carried out in the absence or presence of these PS1-TM peptides. To facilitate the binding of XYT1032 by the peptides, XYT1032 and PS1 peptides were pre-incubated *in vitro* before they were added to the cells. As shown in Figures 6c and d, PS1-TM6 significantly out-competed XYT1032 in the labeling of PS1-NTF in a dose-dependent manner, whereas other PS1-TMs did not (Figure 6e). These results indicate XYT1032 and its parent compounds XYT472B and 3AA may bind to the sixth transmembrane domain of PS1.

Asp257 and Asp385, residing in the sixth and seventh transmembrane domain of PS1, respectively, are critical structural and functional elements in the catalytic cores of γ-secretase [37, 38]. γ-secretase inhibitors inhibit γ-secretase activity by directly binding to its catalytic cores [17, 39]. Interestingly, we did not observe any obvious inhibitory effects of 3AA or XYT472B on γ-secretase (Figures 3h and 4). Furthermore, binding of 3AA or its analogs to the sixth transmembrane domain of PS1 does not interfere with the substrate processing by γ-secretase as monitored with *in vitro* C99 processing assay. As shown in Figure 6f, L-685 458 treatment abolished AICD accumulation, while BACE1 inhibitor-IV and E2012 treatment did not cause any detectable changes in AICD production. Meanwhile, treatment with neither 3AA (Figure 6f, left), nor XYT472B (Figure 6f, right), caused detectable change in AICD production. We then investigated the effects of 3AA and XYT472B on Notch and E-Cadherin, which undergo proteolysis by γ-secretase to acquire their biological activities. HEK293T cells transiently transfected with a myc-NotchΔE construct and a human epidermis cell line (A-431) with detectable endogenous E-Cadherin levels were used to test NICD and E-Cad CTF γ production. Treatment of γ-secretase inhibitors L-685 458 and BMS-708163 greatly inhibited NotchΔE and E-Cad CTF γ processing, leading to significantly reduced levels of NICD (Figure 6g) and E-Cad CTF γ (Figure 6h). 3AA and XYT472B did not have significant effects on NICD (Figure 6g) or E-Cad CTF γ (Figure 6h) accumulation. These substrate-processing patterns we observed indicate that 3AA and XYT472B do not act by directly inhibiting the γ-secrease activity. Interestingly, previous studies have shown that TM6 of PS1 may involve in the initial substrate binding [38]. We then asked if chemical binding of the sixth transmembrane domain of PS1 could be abolished by mutations in this region. We created a set of mutants and carried out photo-click affinity labeling experiments with XYT1032. We found that the labeling of L248/V252/Y256 mutants was attenuated (Supplementary Figure S2a) and the labeling of XYT1032 could not be out-competed by XYT472B (Supplementary Figure S2b), indicating that this region is the possible binding site. These data together suggest that binding of 3AA and XYT472B to the sixth transmembrane domain of PS1 could preferentially modulate APP binding.

Our chemical binding results and substrate-processing patterns indicate that 3AA and XYT472B interfere with PS1/BACE1 interaction by binding to PS1 at a site distinct from the catalytic core of γ-secretase, and targeting PS1/BACE1 interactions with these chemicals do not cause detectable effects on the processing of other γ-secretase substrates.

### XYT472B alleviates cognitive deficits and Aβ pathology in APP/PS1 transgenic mice

**Figure 7:**
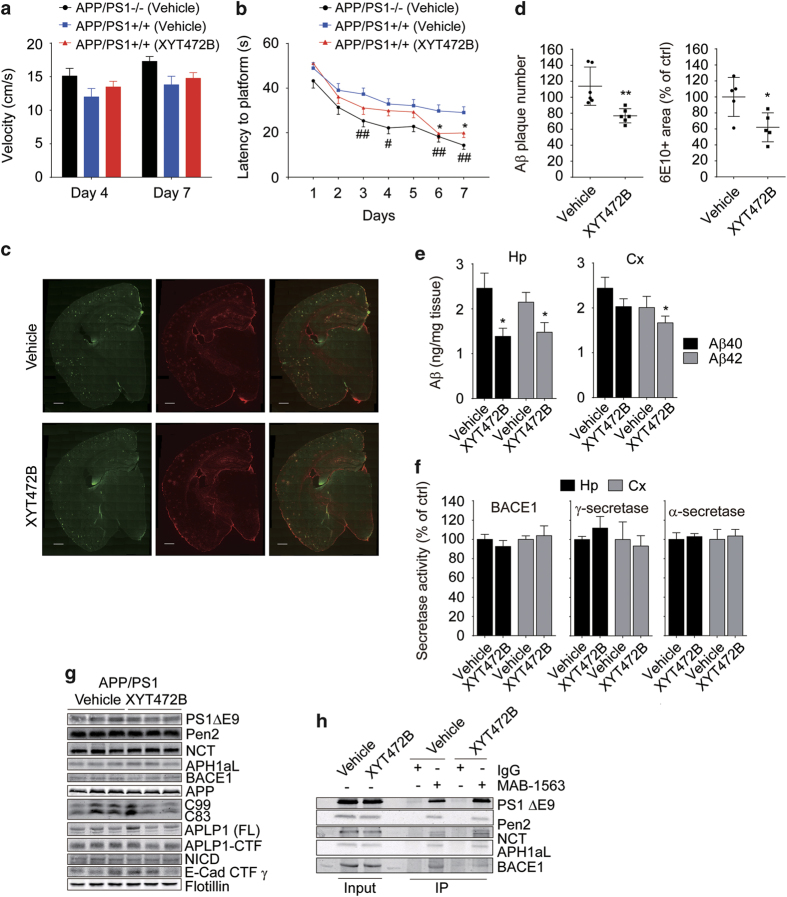
XYT472B attenuates behavior deficits and Aβ pathology of APP/PS1 mice. (**a**) Swimming velocities of mice in all groups during the probe trials on day 4 and day 7. (**b**) XYT472B treatment ameliorates the cognitive deficits of APP/PS1 mice in Morris water maze. APP/PS1 mice show a slower learning curve compared with WT littermates with statistical significance on days 3, 4, 6 and 7. This impairment was partially improved by XYT472B treatment (with statistical significance on days 6 and 7). *n*=16 for each group. ^#^*P*<0.05, ^##^*P*<0.01, *P<0.05, determined by two-way ANOVA. (**c**) Representative brain immunofluorescent images of vehicle- or XYT472B-treated mice. (**d**) Statistical analyses show that 6E10-positive Aβ plaques and GFAP-positive area were reduced by XYT472B treatment. Scale bar, 500 μm. (**e**) Reduced Aβ levels in hippocampus and prefrontal cortex of XYT472B-treated mice. Extracts from hippocampus or cortex were prepared and subjected to sandwich ELISAs for human Aβ40 and Aβ42. (**f**) BACE1, γ-secretase and α-secretase activities were unaltered by chronic treatment of XYT472B. Fluorogenic substrate assays were performed with the membrane fractions extracted from mouse hippocampi or cortices. (**d**–**f**) *P<0.05, **P<0.01, determined by unpaired Student’s *t*-test. (**g**) Chronic XYT472B treatment shows no effects on secretase expression or substrate processing. Extracts from hippocampus were analyzed by western blotting. (**h**) Chronic XYT472B treatment attenuates the interaction between BACE1 and PS1ΔE9 *in vivo*. Vehicle- or XYT472B-treated APP/PS1 mouse brains were homogenized, and purified membrane fractions were incubated with IgG or MAB-1563 antibody for immunoprecipitation. Cx, cortex; Hp, hippocampus.

We then accessed the pharmaceutical efficacy of XYT472B in a transgenic mouse model of Alzheimer's disease. APP/PS1 double-transgenic mice express a chimeric mouse/human APPswe and a human PS1 with exon-9 deletion (PS1ΔE9). These mice display an aggressive onset of age-dependent neuritic Aβ deposition in the cortex and hippocampus [40] and begin to show memory deficits from 6 months of age [41]. We utilized osmotic mini-infusion pumps to achieve a continuous delivery of XYT472B into the mice. At 3 to 3.5 months after birth, age- and gender-matched APP/PS1 transgenic mice and their transgene-negative littermates were subjected to subcutaneous implantation of osmotic pumps filled with vehicle or XYT472B at a daily dose of 10 mg kg^−1^. Ten weeks later, we assessed cognitive function and spatial memory of these mice in a Morris water maze. No obvious adverse effects were observed during compound administration, and we detected no significant difference in swimming speed between APP/PSI mice and control mice in the test (Figure 7a). However, during the hidden platform training, APP/PS1 mice began to show significantly slower learning latencies than their transgene-negative littermates on day 3 (Figure 7b, *P*<0.01, APP/PS1 mice versus wild-type littermates); this difference persisted on days 4, 6 and 7. In contrast, XYT472B-treated APP/PS1 mice showed significantly improved performance than the vehicle-treated APP/PS1 mice on days 6 and 7 (*P*<0.05, APP/PS1 XYT472B-treated mice versus APP/PS1 vehicle-treated mice). These results suggest that XYT472B treatment can alleviate the spatial learning and memory deficits of APP/PS1 mice.

Following the behavioral tests, mice were anesthetized and killed for pathological analysis. APP/PS mice at 6.5 months of age showed detectable Aβ deposits, whereas XYT472B-treated APP/PS1 mice had markedly reduced Aβ deposits in the hippocampal and cortical areas (representative images as shown in Figure 7c, Figure 7d left, *P*=0.0052; Figure 7d, *P*=0.0234, determined by Student’s *t*-test). We next quantified the Aβ40 and Aβ42 levels of these mice by sandwich ELISA. In the hippocampus of XYT472B-treated mice, both Aβ40 and Aβ42 levels were significantly reduced (Figure 7e, right, *P*=0.0211 for Aβ40 and *P*=0.0461 for Aβ42). Similar results were obtained in the prefrontal cortices, where Aβ40 and Aβ42 levels were reduced by roughly 20% (Figure 7e, left, *P*=0.08 for Aβ40, *P*=0.0389 for Aβ42). These results indicate that administration of XYT472B attenuates Aβ pathology and reduces *in vivo* Aβ loading in APP/PS1 transgenic mice.

We also examined the secretase activities in the XYT472B-treated mice using fluorogenic substrate assays and found no discernible difference in BACE1, γ-secretase or α-secretase (Figure 7f) activitiy where compared with the vehicle-treated APP/PS1 mice. Moreover, we observed no obvious changes in the expression levels of the secretase components (Figure 7g). In addition, we monitored the steady-state processing of secretase substrates: NICD, E-Cadherin CTF-γ and APLP1 (amyloid-like protein 1) fragments in a western blot analysis. As shown in Figure 7g, levels of APP, C83 and C99, as well as NICD, E-Cad CTF-γ and APLP1-CTF were also indistinguishable among vehicle- or XYT472B- treated APP/PS1 mice.

Finally, we examined the endogenous interaction between γ-secretase and BACE1 in vehicle- or XYT472B-treated mouse brain samples. As shown in Figure 7h, immunoprecipitation of both vehicle- and XYT472B-treated APP/PS1 mouse brain samples with MAB-1563, an antibody recognizing PS1-N-terminus, pulled down PS1ΔE9, Pen2, NCT and APH1aL. Also as expected, BACE1 was precipitated in the sample from vehicle-treated mice, demonstrating the endogenous interaction between BACE1 and γ-secretase. But in the brain sample from XYT472B-treated mice, significantly less BACE1 was detected in the immunoprecipitates, confirming that XYT472B treatment attenuated the interaction between BACE1 and γ-secretase.

Altogether, these results indicate that XYT472B treatment can ameliorate Aβ-related pathology without affecting the secretase activities and improve the cognitive function of APP/PS1 mice.

## Discussion

**Figure 8:**
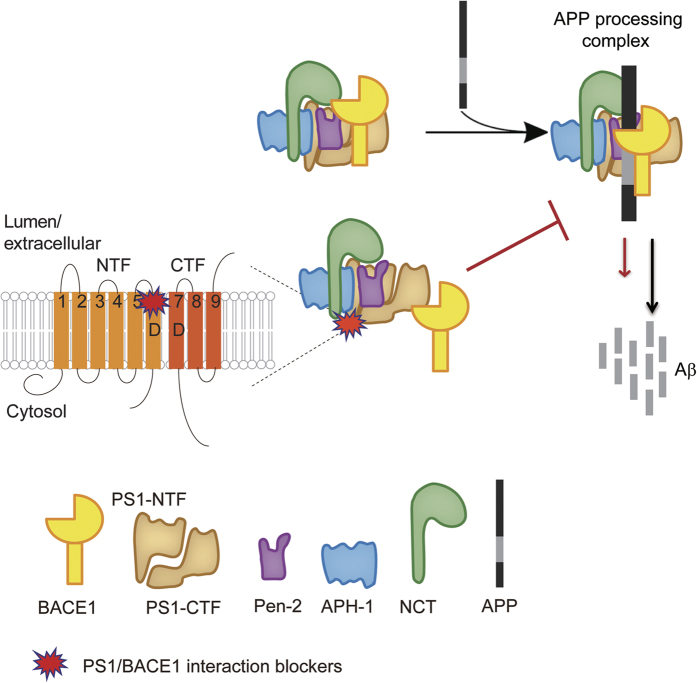
A working model of 3AA and XYT472B. 3AA and XYT472B (PS1/BACE1 interaction blockers) bind to the sixth transmembrane domain of PS1, interfere with the β-/γ-secretase interaction and reduce Aβ production.

In this study, we demonstrate that the small molecule 3AA and its structural analog XYT472B can block the interaction between PS1 and BACE1, and reduce Aβ production. Further, an XYT472B-derived photoclickable probe, XYT1032, binds directly to the PS1 subunit of the γ-secretase complex. Binding of XYT1032 was abolished by its parent compounds 3AA and XYT472B, but not by L-685 458, indicating that 3AA and XYT472B modulate PS1/BACE1 interaction by binding to PS1 outside of the catalytic core of γ-secretase. Moreover, 3AA and XYT472B enhanced the labeling of an E2012-derived probe to PS1-NTF and Pen2, suggesting 3AA and XYT472B may potentiate the binding of E2012 to PS1. In contrast, binding of L-685 458 had no obvious effect on the binding of E2012 to PS1. These results further support the idea that 3AA and XYT472B may bind to PS1 at a site different from the catalytic core. Of note, despite the significant reduction of Aβ by 3AA and XYT472B, we didn’t observe any obvious inhibitory effects of either 3AA or XYT472B on BACE1 and γ-secretase activities, suggesting that instead of directly modulating individual catalytic activity of the secretases, 3AA and XYT472B block PS1/BACE1 interaction and hence slow down the sequential processing of APP by each enzyme (Figure 8). Interestingly, SPI-1865, a newly discovered triterpene class of GSMs originally extracted from Black Cohosh [42–44], possesses a similar chemical structure to that of our lead compound 3AA. It remains to be investigated whether these compounds operate similarly. Should this be the case, 3AA and XYT472B along with these chemicals might be categorized as a novel class of GSM compounds that display unique pharmacological properties due to their ability to interfere with the PS1/BACE1 interaction, in contrast to conventional secretase inhibitors or modulators.

BACE1 has maximal catalytic activity at low pH, consistent with its primary localization in endosomes, lysosomes and trans-Golgi network [45]. γ-secretase assembly initiates in the endoplasmic reticulum, and γ-secretase is active at the cell surface, and in early to late endosomes and lysosomes [46–48]. Endocytic pathway is crucial in the amyloidogenic processing of APP [49, 50], which requires that both secretases are transported into endosomal environment and APP are internalized from the plasma membrane via clathrin and/or raft-dependent endocytosis [49, 51]. Together with our co-localization data, we speculate that PS1/BACE1 interaction may occur in the endosomal environment, which is more suitable to APP processing. Our previous studies showed that some G-protein-coupled receptors, including β_2_ adrenergic receptor and delta opioid receptor, regulate γ-secretase and BACE1 activity by modulating the endocytosis of secretases in an interaction-based manner [22, 23]. Thus it would be of interest and importance to investigate whether these secretases, receptors and other modulators form a larger protein complex to fine-tune APP processing, which should provide other potential therapeutic targets with less side effects.

Decades of efforts have been invested in developing anti-AD drugs by either inhibiting or modulating γ-secretase or BACE1 activities. Unfortunately, most of these molecules failed in clinical trials because of the severe side effects associated with substrate processing in peripheral tissues [36, 52, 53]. Considering these daunting obstacles in the development of secretase inhibitors and modulators, targeting protein–protein interactions may provide a novel alternative for the therapeutic intervention of AD. Our report presents small molecules 3AA and XYT472B as Aβ generation modulators, operating by interfering with the PS1/BACE1 interaction. Additional compounds with similar properties are required to fully establish the therapeutic potential of this promising approach.

## Materials and Methods

### Chemicals

3AA with 98% purity was purchased from BioBioPha (Kunming, China). Synthesis of XYT472B and XYT1032 is described in the Supplementary Information. L-685 458 was purchased from Sigma (St Louis, MO, USA) and BACE1 inhibitor-IV (BSI-IV) was purchased from Calbiochem (Hayward, CA, USA). BMS-708163 (BMS) and LY-2886721 (LY) were purchased from Selleck Chemicals (Houston, TX, USA). E2012 and E2012-Bpyne were synthesized by Ginkgo Pharma (Suzhou, China).

### Antibodies

Immunoblotting and immunostaining were performed with the following antibodies: anti-PS1 N (1–65) (529591, EMD anti-PS1, dilution 1:3 000, EMD Millipore, Darmstadt, Germany); anti-PS1 loop (263–407; 529592, dilution 1:2 000, Calbiochem); anti-Pen2 (P5622, dilution 1:1 000, Sigma); anti-NCT (N1660, dilution 1:1000, Sigma); anti-Aph1aL/C-terminal (245–265; PRB-550P, 1:1000, Covance, Davis, CA, USA); anti-BACE1 N-term (AP7774b, dilution 1:1000, Abgent, Suzhou, China); anti-APP-CTF (A8717, dilution 1:1000, Sigma); anti-Flag (F3156, dilution 1:2000, Sigma); anti-sAPPβ (6A1; 10321, dilution 1:1000, IBL, Hokkaido, Japan) anti-HA (H6908, dilution 1:5 000, Sigma); anti-c-Myc (9E10; sc-40, dilution 1:500, Santa Cruz Biotechnology, Santa Cruz, CA, USA); anti-NICD (D3B8; 4147, dilution 1:1000, Cell Signaling Technology, Beverly, MA, USA); anti-E-Cadherin-CTF (610182, dilution 1:1 000, BD Transduction Laboratories, San Jose, CA, USA); anti-APLP-1 C-Terminal (643–653; 171615, dilution 1:1000, Calbiochem); anti-Aβ 40 clone G2–10 (MABN11, dilution 1:1000, EMD Millipore); anti-Beta Amyloid, 1–16 (Monoclonal 6E10; SIG39300, dilution 1:1000, Covance); anti-calnexin (ab22595, 1:500, abcam, Cambridge, MA, USA); GM130 (610822, dilution 1:200, BD transduction laboratories); or EEA1 (610457, dilution 1:200, BD transduction laboratories); anti-GFAP (z0334, dilution 1:500, Dako, Aichi-ken, Japan).

### Plasmids

cDNA sequences of human Aph1aL, Nct, PS1 and Pen2 were codon-optimized and cloned into pcDNA3 vector to generate pAph1aL, pNct, pPS1 and pPen2 plasmids with varying tags (Life Technologies, Grand island, NY, USA). Plasmids PS1-NTF-NTEV, PS1-NTF-CTEV, BACE1-NTEV, BACE1-CTEV, APH1aL-CTEV, APH1aL-NTEV were generated by sub-cloning the cDNA sequences of these genes into p3639 vector, followed by ligation with -NTEV or -CTEV tags (Figure 1j). Each protein is tagged with NTEV or CTEV with a 12 amino acid linker, GSGGGGSGGGGS, in between. For FRET constructs, CFP or YFP was fused to the N-terminus or C-terminus of each protein with the same linker and cloned into p3639 vector. We performed site-directed mutagenesis using an overlapping PCR strategy, with wild-type PS1 construct as template, and subcloned the mutant into pcDNA3 vector. p3639, NTEV-KvBeta1, Kv1.1-CTEV, ERT2-tev-LexA-Gal4 and LexA-op-F-lucF-luciferase constructs were provided by Sanofi-Aventis Research and Development (Paris, France).

### Cell culture

HEK293T, HEK293MSR, HEK293/APPswe, U-2 OS and A-431 were cultured in Dulbecco’s modified Eagle’s medium with 10% (w/v) heat-inactivated fetal bovine serum in a humidified incubator with 5% CO_2_/95% air (v/v) at 37 °C. HEK293 cells were cultured in MEM under the same condition.

### Immunofluorescence microscopy

U-2 OS cells cultured on glass cover slips were transiently transfected with designated constructs using Fugene HD Transfection Reagent (Roche, Basel, Switzerland). Twenty-four hours after transfection, cells were fixed 4% paraformaldehyde in phosphate-buffered saline (PBS), permeabilized and blocked with PBS containing 0.2% Triton X-100 and 1% bovine serum albumin. Cells were next incubated with primary antibodies against calnexin, GM130 or EEA1. After washing in PBS containing 1% bovine serum albumin, cells were incubated with Cy3- or Cy5-labeled goat anti-mouse or rabbit IgG secondary antibodies in the dark. Cells were washed again and finally mounted on slides. Images were acquired on a LAS SP8 confocal microscope (Leica, Wetzlar, Germany) with a 63×/1.40 NA oil objective (Leica). Floating coronal ice sections of the mouse brains (30 μm thick) were incubated with antibodies against Aβ plaques (6E10) and astrocytes (GFAP) at 4 °C for 12 h. Images were captured using an AxioVert inverted fluorescence microscope (Carl Zeiss, Oberkochen, Germany) and quantification was performed using Image-Pro Plus 5.1 software (Media Cybernetic, Rockville, MD, USA).

### Acceptor photobleaching FRET assay

HEK293 cells were cultured on glass coverslips and transfected with Effectene Transfection Reagent (QIAGEN, Hilden, Germany). After fixing in 2% paraformaldehyde in PBS for 30 min, cells were washed in PBS three times and mounted on slides. Samples were subjected to acceptor photobleaching FRET imaging with a confocal microscope (LAS SP8; Leica) with a 63×/1.40 NA oil objective (Leica). Image acquisition, registration, background subtraction and data analyses were performed with Leica Application Suite Advanced Fluorescence (LAS AF) software. Imaging conditions were set up manually: CFP (excitation: 405 nm, emission: 465–505 nm) and YFP (excitation: 514 nm, emission: 525–600 nm). Photobleach was performed using 514-nm light and over 70% bleach efficiency was achieved. Images of CFP and YFP channels were acquired pre- and post-bleach. FRET efficiency was calculated as percentage of enhancement in donor fluorescence (f) after acceptor photobleaching: E=1-f(CFP_(pre)_)/f(CEP_(post)_). Five non-bleached regions were selected and the average values were used to correct the FRET efficiency of photobleached region.

### Split-TEV assay

About 1×10^6^ HEK293MSR cells were seeded in each well in 24-well plates, or 2×10^4^ cells per well in 96-well plates. In 24-well plates, each well of HEK293MSR cells were transiently transfected with total 0.8 μg DNA containing equal molar ratio of -NTEV and -CTEV fusion-protein constructs, ERT2-tev-LexA-Gal4, LexA-op-F-lucF-luciferase, and 15 ng hRluc as internal control. For 96-well plates, 125 ng DNA containing equal molar ratio of -NTEV and -CTEV fusion-protein constructs, ERT2-tev-LexA-Gal4 and LexA-op-F-lucF-luciferase constructs, was transfected in each well. Two hours after the transfection, chemicals were added. Cells were treated for 16 h before the measurements of luciferase activity.

### Co-immunoprecipitation assay

Co-immunoprecipitation assays of γ-secretase complex with BACE1 were modified according to a previous report [54]. Thirty-six hours after transient transfection, HEK293T cells were treated with designated chemicals or peptides for 16 h before collection. Cells were washed twice with cold PBS and lysed with IP buffer (50 mM HEPES pH 7.4, 150 mM NaCl, 10% Glycerol and 1% CHAPSO), in the absence or presence of chemicals or peptides. After centrifugation, the supernatants were incubated with anti-Flag M2 resins at 4 °C for 4 h. The resins were washed three times and eluted with SDS loading buffer before western blotting analysis. Half brains of vehicle- or XYT472B-treated APP/PS1 mice were homogenized by a glass dounce tissue grinder in buffer A (25 μM Tris-HCl, 5 mM EDTA, 5 mM EGTA, adjusted to pH 7.4) and centrifuged to remove debris and nuclei. After centrifugation at 25 000 *g* for 1 h, 600 mg membrane proteins were resuspended in IP buffer and incubated with antibodies at 4 °C for 16 h. For each mouse sample, equal amount of membrane fractions were incubated with 2 μg goat anti-rat IgG or MAB-1563. The antibody-antigen complexes were then incubated with pre-equilibrated Ezview Red Protein G Affinity Gel beads (Sigma) for 1 h at 4 °C. After washed, the resins were eluted with SDS loading buffer for western blotting analysis.

### ELISA for Aβ

HEK293/APPswe cells were seeded in 96-well plates and treated with chemicals of designated concentrations and time. Cell media were then collected and subjected to sandwich ELISA. Human Aβ40 and Aβ42 in APP/PS1 mouse brains were extracted as previously reported [22] and measured with human Aβ ELISA kits. ELISA kits for total human Aβ, human Aβ40 and Aβ42 were obtained from ExCell Bio (Shanghai, China). ELISA kits for human Aβ38 were purchased from IBL International.

### *In vitro* BACE1 and γ-secretase assays

Total membrane fractions were extracted from HEK293T cells and used for fluorogenic substrate assays to measure BACE1 or γ-secretase activity, or ELISA-based γ-secretase activity assays. Fluorogenic substrate assays were carried out as previously reported [55, 56]. For ELISA-based γ-secretase activity assay, HEK293T cells were lysed in buffer A and centrifuged to remove nuclei and large cell debris. The supernatants containing 120 μg protein were centrifuged at 25 000 *g* for 1 h. The resulting membrane pellets were resuspended in γ-assay buffer (0.25 % CHAPSO, 20 mM HEPES pH 6.8, 150 mM KCl, 2 mM EDTA and 0.1 μg μl^−1^ biotinylated APP-TM peptide). After incubation at 37 °C for 2 h, the reaction mixtures or biotinylated standard peptides p-40 were added into streptavidin-coated 96-well plates (Pierce, Life Technologies, Grand Island, NY, USA) and incubated at room temperature for 1 h. After washes, the plates were incubated with anti-Aβ40 antibodies (clone G2–10, EMD Millipore) for 1 h and washed again. Next, horseradish peroxidase-labeled anti-mouse antibodies were added and the plates were incubated for 1 h. Ultra-TMB (Pierce) was used as the substrate of horseradish peroxidase and the absorbance at 450 nm was recorded. Concentrations of samples were calculated according to standards. APP-TM peptide amino acid sequence: biotin-KGAIIGLMVGGVVIATVIVITLVMLKKK-Amide, p-40 amino acid sequence: biotin-KGAIIGLMVGGVV (APP-TM and p-40 were synthesized by Glbiochem, Shanghai, China).

### Photo-affinity labeling and competition in live cells

Photo-affinity labeling and competition experiments were carried out as previously reported [35], with modifications. Forty-eight hours after transient transfection, HEK293T cells were incubated with XYT1032 with/without competing chemicals at 37 °C in 5% CO_2_ for 2 h, and UV-irradiated (365 nm) for 30 min. Cells were washed three times with cold PBS and lysed in IP buffer (50 mM HEPES pH 7.4, 150 mM NaCl, 1% CHAPSO or 1% Triton X-100 and protease inhibitor cocktails). The lysates were incubated with click chemistry reagents (1 mM CuSO4, 10 mM L-Ascorbic acid, 3 μM biotin alkyne for XYT1032 labeling or 1 μM biotin azide for E2012-Bpyne labeling) at room temperature for 1 h. The clicked proteins were ultracentrifuged with Centricon (10 kD, Merck Millipore, Billerica, MA, USA) and washed in cold IP buffer twice to remove the residual click reagents, affinity enrichment with streptavidin resins or anti-Flag M2 resins (Figure 3d) and western blotting analysis.

### γ-secretase substrate-processing assays

HEK293/APPswe cells or HEK293T cells transiently transfected with myc-NotchΔE were treated with chemicals for 4 h and membrane fractions or total lysates were analyzed for the APP-CTFs and NICD levels. Culture media of HEK293/APPswe cells were subjected to western blotting analysis for sAPPβ. *In vitro* C99 assay was carried out as described previously[57]. Membrane fractions of A-431 cells were incubated with chemicals *in vitro* at 37 °C for 4 h and then subjected to western blotting for E-Cadherin processing fragments. Membrane fractions of mouse hippocampus were extracted and subjected to western blotting analysis for γ-secretase components: BACE1, APP and CTFs, APLP1 and CTF, NICD and E-Cadherin CTFs. Actin or flotillin was blotted as a control.

### C99-GVP and NotchΔE-GVP reporter assay

We used HEK293T cells for this assay followed previously reported protocol [58]. Luciferase activities were measured using Dual-Luciferase Reporter Assay System (Promega, Madison, WI, USA).

### siRNA-mediated knockdown of Bace1 expression

An siRNA-mediated knockdown approach was utilized [59, 60]. siRNA targeting human *Bace1* (5′-GCUUUGUGGAGAUGGUGGA-3′, GenePharma, Shanghai, China) was transfected into HEK293T cells and 72 h later, cells were harvested for real-time qPCR analysis of *Bace1* expression and *in vitro* C99 assay. Primer sequences used for *Bace1* expression analysis are: 5′-CCCGAAAACGAATTGGCTTT-3′ and 3′-GCTGCCGTCCTGAACTCATC-5′.

### Cell viability test

Chemically treated HEK293/APPswe cells were washed twice with PBS and subjected to CellTiter-Glo Luminescent Cell Viability Assay (Promega) following guidance of the manufacturer.

### Animals and chemical administration by osmotic pump implantation

All animal experiments were performed according to the National Institutes of Health Guide for the Care and Use of Laboratory Animals. Animal protocols were approved by the biological research ethics committee, Shanghai Institutes for biological Sciences, Chinese Academy of Sciences. Animal pain and discomfort were minimized with efforts. APPswe/PS1ΔE9 (APP/PS1) double-transgenic mice (The Jackson Laboratory, Bar Harbor, ME, USA, stock number 004462) were maintained and genotyped according to the guidance of Jackson Laboratory. Transgene-negative wild-type littermates were used as age-matched controls. At 3.5 months, age- and gender-matched APP/PS1 and wild-type mice were anesthetized and subjected to subcutaneous implantations of osmotic pumps (Model 2006, Alzet, Cupertino, CA, USA) that delivered a daily dose of 10 mg kg^−1^ (*n*=16 for each group). Littermates were evenly divided into a vehicle-treated group and a XYT472B-treated group. XYT472B dissolved in 50% dimethyl sulfoxide and 50% ethylene glycol was loaded into the pumps. Pumps filled with 50% dimethyl sulfoxide and 50% ethylene glycol served as the vehicle control. Six weeks later, the first pumps were removed and replaced by the second ones.

### Morris water maze

Morris water maze was performed by investigators blinded for mice genotypes and groups, as previously reported [61]. In brief, the apparatus was a circular pool of 120 cm diameter filled with water containing small white plastic particles and cues of four different shapes posted on four directions of the inner pool wall. The water temperature was maintained at 23.0±0.5 °C and the room temperature at 25.0±0.5 °C during the whole experiment. A transparent platform 11 cm in diameter was placed 0.8 cm below the water surface at a fixed position in one quadrant. The training lasted 7 consecutive days, with four trials per day. On days 4 and 7, probe trials were performed after the training trials. An automated tracking system (Ethovision XT software by Noldus Information Technology, Wageningen, Netherlands) was used to monitor the mouse swimming paths.

### Statistical analysis

All experiments were repeated at least three times. All data are presented as mean±s.e.m. and analyzed by GraphPad Prism 6.01 (San Diego, CA, USA). Unpaired Student’s *t*-test was applied for comparisons of two groups. Group differences were analyzed with one-way analysis of variance following Bonfferoni’s multiple comparison test. The results of Morris water maze hidden platform training were compared using two-way analysis of variance. Differences were considered significant when *P*<0.05.
