# Supplementary figures and images for: Targeting the γ-/β-secretase interaction reduces β-amyloid generation and ameliorates Alzheimer’s disease-related pathogenesis
Source: Cell Discov. 2015 Aug 18;1:15021–. doi: 10.1038/celldisc.2015.21 (PMC4860824; doi:10.1038/celldisc.2015.21)

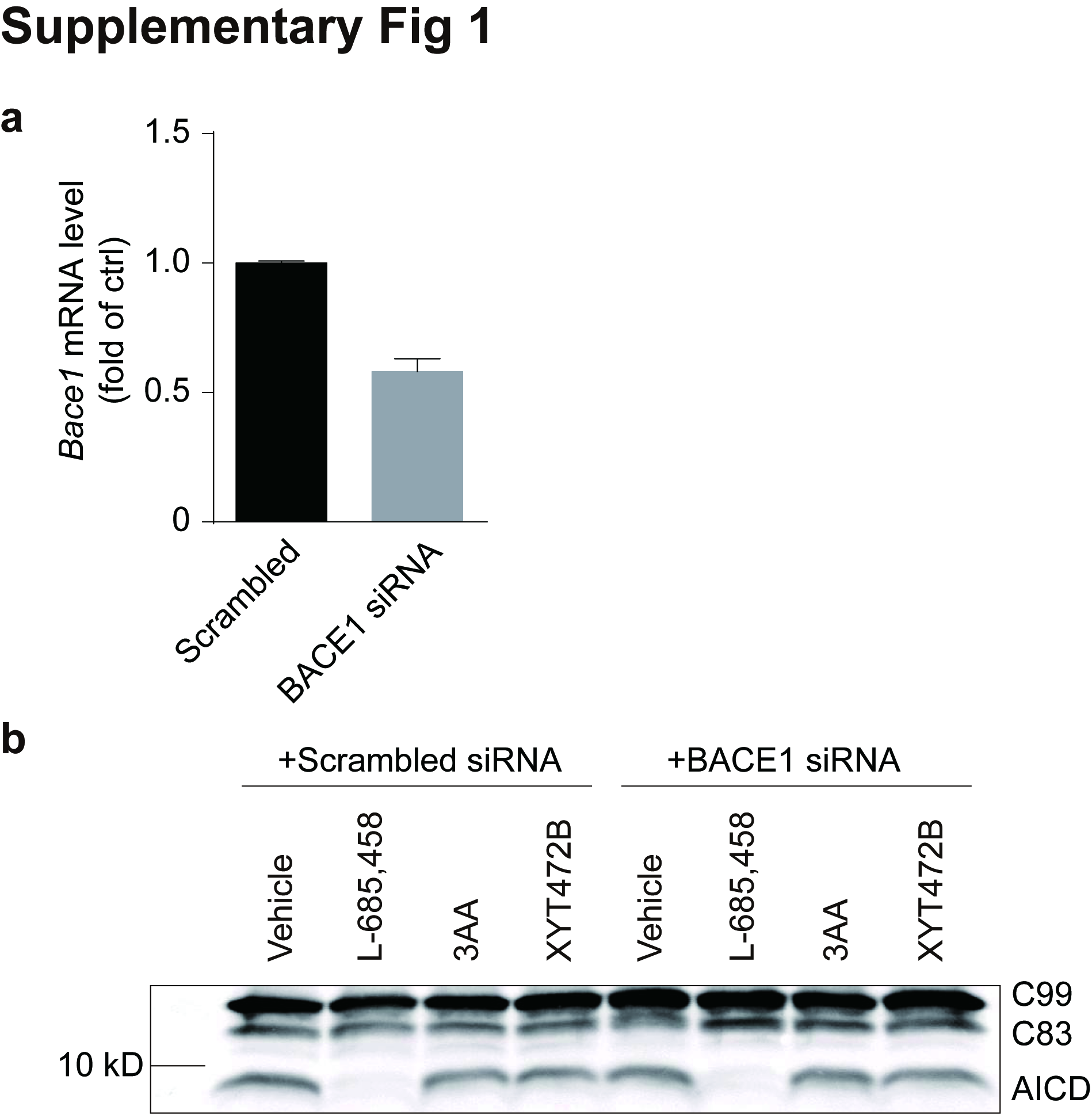

Supplement: Supplementary Figure S1 [file celldisc201521-s1.tiff]

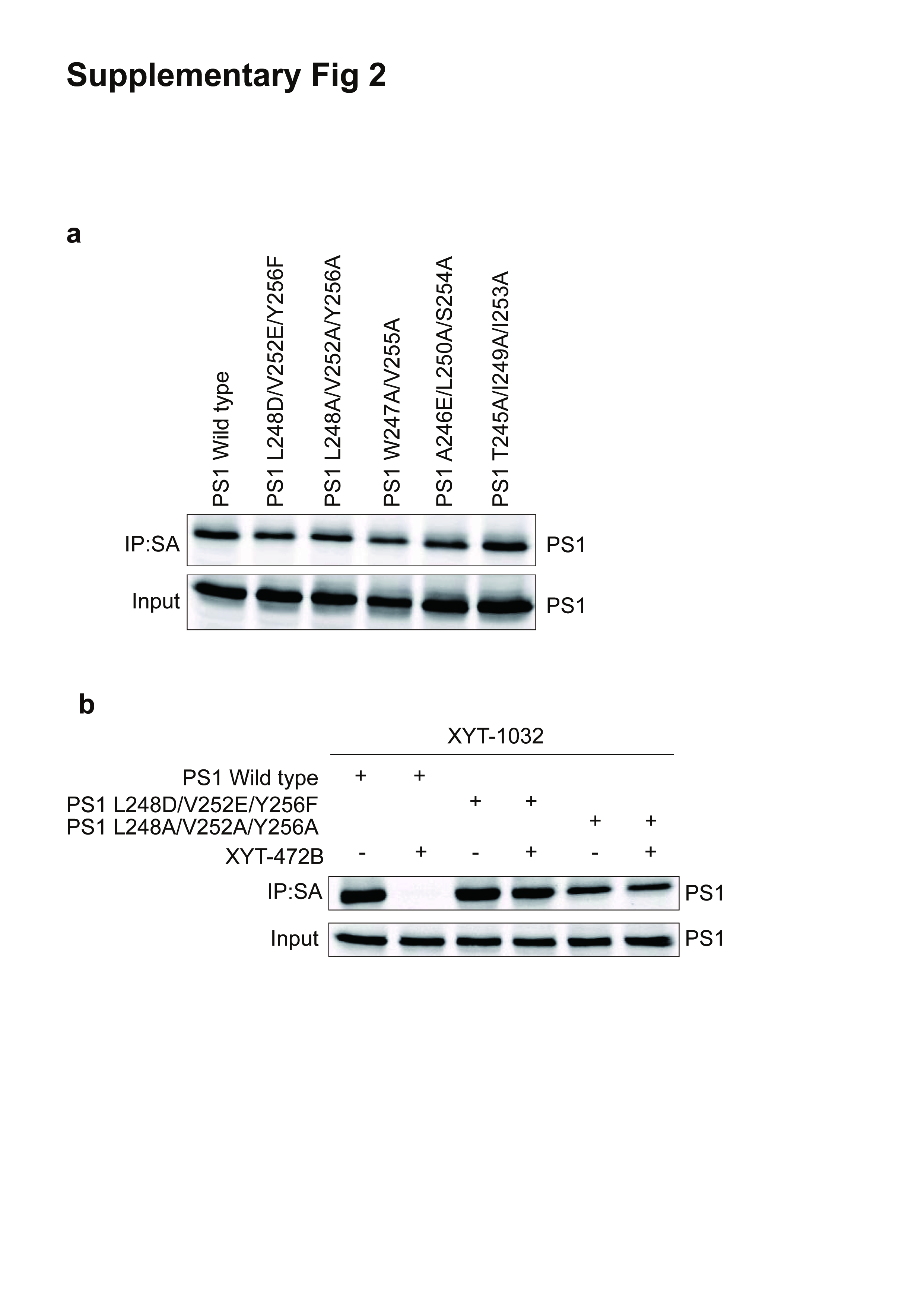

Supplement: Supplementary Figure S2 [file celldisc201521-s2.tiff]
