# Supplementary material for: Targeting the γ-/β-secretase interaction reduces β-amyloid generation and ameliorates Alzheimer’s disease-related pathogenesis
Source: Cell Discov. 2015 Aug 18;1:15021–. doi: 10.1038/celldisc.2015.21 (PMC4860824; doi:10.1038/celldisc.2015.21)
Supplement: Supplementary Information [file celldisc201521-s3.pdf]

Supplementary Table 1. Small molecule screening data

Category	Parameter	Description
Assay	Type of assay	Cell-based
	Target	PS1/BACE1 interaction
	Primary measurement	Detection of Firefly luciferase enzyme activities induced by PS1-NTF-NTEV/BACE1-CTEV interaction.
	Key reagents	SteadyGlo Reagent (Promega)
	Assay protocol	Bulk transfection was carried out with the suspended HEK293MSR cells after trypsinized. Then 2x104 cells were seeded to each well of 96-well plates. For each plate, 12 μg total DNA was transfected with Fugene HD reagent (Roche), with equal molar ratio of PS1-NTF-NTEV and BACE1-CTEV fusion protein constructs, ERT2-tev-LexA-Gal4, LexA-op-F-lucF-luciferase. As a control, same experiments were carried out with NTEV-KvBeta1 and kv1.1-CTEV transiently transfected to the cells. Two hours after transfection and seeding, cells were treated with 10 μM small molecules. The luciferase enzyme activities were determined following 18 h treatment. were determined for Wnt signaling activity
	Additional comments	The positive small molecule only impacted luciferase enzyme activity induced by PS1-NTF/BACE1 interaction group but not that of KvBeta1/Kv1.1 group.
Library 1	Library size	1,281
	Library composition	Pharmacologically-Active Sigma Compounds
	Source	Lopac (Sigma)
	Additional comments	No.
Library 2	Library size	1,440
	Library composition	Natural products
	Source	BioBioPha Co., Ltd
	Additional comments	No.
Screen	Format	96-well, Perkin Elmer 6005680
	Concentration(s) tested	10 μM compound, 1% DMSO
	Plate controls	1% DMSO
	Reagent/ compound dispensing system	DMSO
	Detection instrument and software	EnVision (PerkinElmer)
	Assay validation/QC	Standard deviation of controls ≤ 15%
	Correction factors	No.
	Normalization	No.
	Additional comments	No.
Post-HTS analysis	Hit criteria	PS1-NTF-NTEV/BACE1-CTEV interaction induced

		luciferase activity changes > 2 standard deviations from the mean of vehicle control group, and NTEV- KvBeta1 and kv1.1-CTEV interaction induced luciferase activity changes < standard deviations.
	Hit rate	Approximately 0.4%,
	Additional assay(s)	Cellular Aβ production and cell viability of HEK293APPswe cells. Acceptor photobleaching FRET assay and over-expressed co-immunoprecipitation of PS1 and BACE1.
	Confirmation of hit purity and structure	Compounds were verified analytically.
	Additional comments	No.

Supplementary Notes

Supplementary Scheme 1

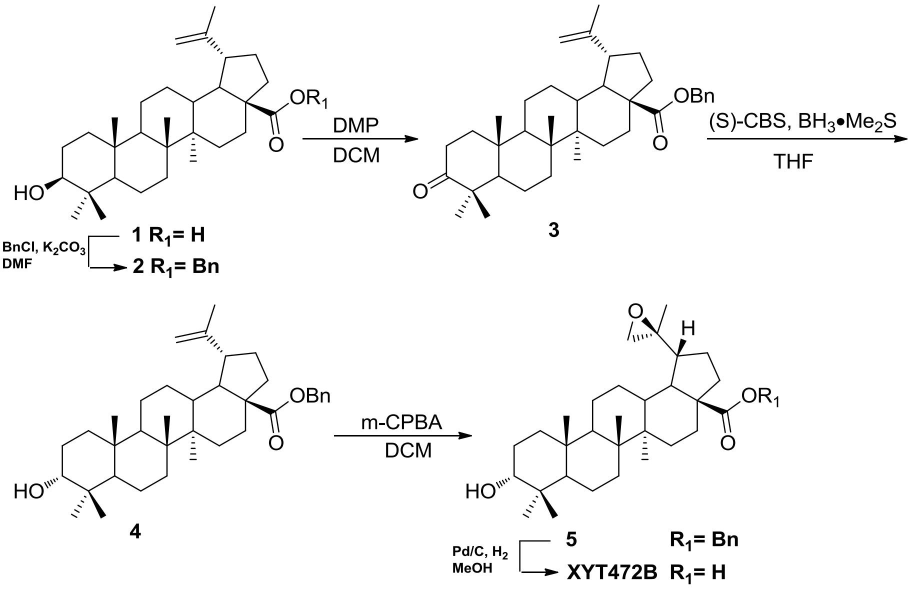

The Synthesis of XYT472B

Benzyl 3 (β)-hydroxylup-20 (29)-en-28-oate (2)

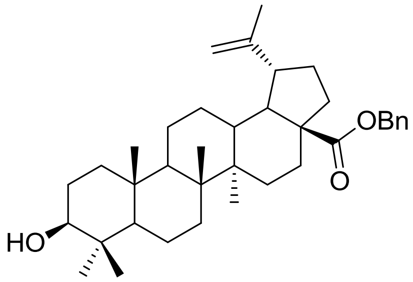

To a solution of betulinic acid **1** (4 g, 8.76 mmol) in DMF (50 mL) was added K_2CO_3 (2.4 g, 17.37 mmol)and benzyl chloride (1.2 mL, 10.52 mmol). The reaction mixture was stirred at 50 ℃ overnight. The mixture was cooled to room temperature, slowly diluted with H_2O (100 mL) and extracted with EtOAc (2×100 mL). The combined organic layers were washed with H_2O (2×150 mL), brine (150 mL), dried over Na_2SO_4 and evaporated under reduced pressure to obtain the desired compound **2** (4.62 g, 8.46 mmol, 97%) as a white solid, which was used for the next step without further purification. ^1H NMR (300 MHz, $CDCl_3$) δ 7.34 (m, 5H), 5.09 (d, 1H, J=11.7 Hz), 5.17 (d, 1H, J=11.7 Hz), 4.75 (s, 1H), 4.62 (s,1H), 3.21–3.15 (m, 1H), 2.92–2.80 (m, 1H), 2.10–1.90 (m, 2H),1.87–1.69 (m, 2H), 1.64 (s, 3H), 1.64–0.96 (m, other aliphatic ring protons), 1.04 (s, 3H), 1.00 (s, 3H), 0.94 (s, 3H), 0.91 (s, 3H),0.87 (s, 3H), 0.84 (s, 3H). ESI-MS (*m/z*): 547.4 $(M+H)^+$.

Benzyl 3-carbonylup-20(29)-en-28-oate (3)

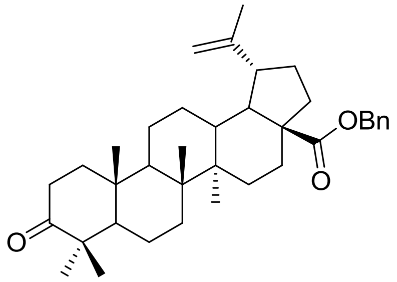

To a solution of compound **2** (4.62 g, 8.46 mmol) in DCM (100mL) was slowly added Dess-Martin Periodinane (4.3 g, 10.15 mmol) at 0 ℃. The mixture was stirred at ambient temperature overnight. The suspension was filtered and the filtrate was concentrated and purified by flash chromatography using 5% EtOAc in petroleum ether to yield 4.39 g (8.06 mmol, 95%) of **3** as a white pure solid. ^{1}H NMR (300 MHz, $CDCl_3$) δ 7.34 (m, 5H), 5.09 (d, 1 H, J=11.7 Hz), 5.17 (d, 1 H, J=11.7 Hz), 4.75 (s, 1H), 4.62 (s, 1H), 2.92–2.80 (m, 1H), 2.49–2.39 (m, 2H), 2.10–2.04 (m, 2H), 1.92–1.80 (m, 2H), 1.78–1.68 (m, 2H), 1.65 (s, 3H),1.50–1.16 (m, other aliphatic ring protons), 1.04 (s, 3H), 1.00 (s, 3H), 0.94 (s, 3H), 0.91 (s, 3H), 0.87 (s, 3H), 0.84 (s, 3H). ESI-MS (*m/z*):567.3 $(M+Na)^+$.

Benzyl 3 (α)-hydroxylup-20 (29)-en-28-oate (4)

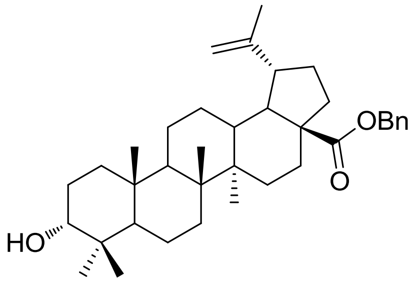

To a solution of compound **3** (1.58 g, 2.90 mmol) was added (*S*)-(–)-2-methyl-CBS-oxazaborolidine (80 mg, 0.29 mmol) in dry THF (50 mL) followed by dropwise addition of a solution of BH_3-Me_2S (0.32 mL, 10 mol/L in THF) at ambient temperature under nitrogen atmosphere. After 10 min, MeOH was added to quench the reaction, and the reaction mixture was concentrated and purified by flash chromatography using 5% EtOAc in petroleum ether to yield 790 mg (1.45 mmol, 50%) of **4** as a white pure solid. ^{1}H NMR (300 MHz, $CDCl_3$) δ 7.34 (m, 5H), 5.09 (d, 1H, J=11.7 Hz), 5.17 (d, 1H, J=11.7 Hz), 4.73 (s, 1H), 4.60 (s, 1H), 3.39 (s, 1H), 3.02–2.96 (m, 1H), 2.28–2.16 (m, 2H), 1.98–1.95 (m,2H), 1.68 (s, 3H), 1.64-0.96 (m, other aliphatic ring protons), 1.04 (s, 3H), 1.00 (s, 3H), 0.94 (s, 3H), 0.91 (s, 3H), 0.87 (s, 3H), 0.84 (s, 3H). ESI-MS (*m/z*): 547.4 $(M+H)^+$.

Benzyl 3(α)-hydroxylup-20(29)-epoxy-28-oate (5)

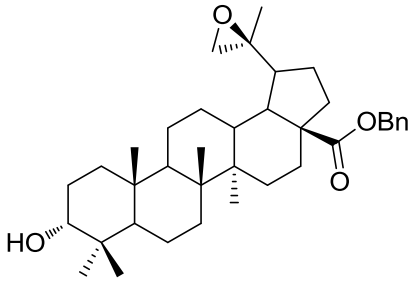

To a solution of compound **4** (1.23g, 2.25 mmol) was added m-CPBA (820 mg, 4.50 mmol) in DCM (20 mL) at ambient temperature under nitrogen atmosphere. After three hours, saturated Na_2SO_3 solution was added to quench the reaction. The mixture was extracted with DCM (2×100 mL). The combined organic layers were washed with H_2O (2×100 mL), saturated $NaHCO_3$ solution (2×100 mL) and brine (150 mL), dried over Na_2SO_4, concentrated and purified by flash chromatography using 25% EtOAc in petroleum ether to yield 1.1 g (1.96 mmol, 87.1%) of **5** as a single configuration which was confirmed by X-ray crystallographic analysis. ^{1}H NMR (300 MHz, $CDCl_3$) δ 7.33 (m, 5H) 5.10 (s, 2H) 3.39 (s, 1H), 2.62 (t, 2H), 2.28 (m, 2H), 2.16 (m, 2H), 1.95 (m, 2H), 1.80 (m, 2H), 1.76-1.32 (m, other aliphatic ring protons), 1.26 (s, 3H), 0.95 (s, 3H), 0.92 (s, 3H), 0.82 (s, 6H), 0.75 (s, 3H). ESI-MS (*m/z*): 585.4 $(M+Na)^+$.

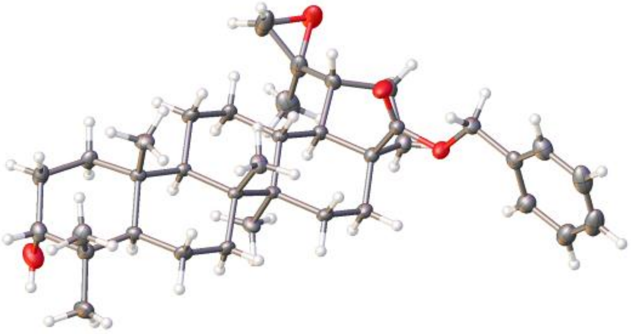

The X-ray crystallographic structure of compound 5

3 (α)-hydroxylup-20 (29)-epoxy-28-oic acid (XYT472B)

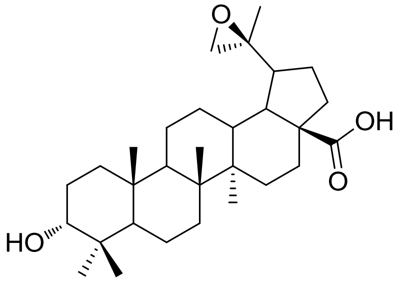

To a stirred solution of **5** (100 mg, 0.18 mmol) in MeOH (2 mL), Pd/C (10 mg) was added at ambient temperature under nitrogen atmosphere. The nitrogen atmosphere was replaced by an H_2 atmosphere. The reaction mixture was stirred for 1 h at room temperature, then N_2 was replaced, and it was filtered through Celite and washed with DCM. The residue was purified by flash chromatography using 10% EtOAc in petroleum ether to yield 51mg (0.11mmol, 61%) of **XYT472B** as a white pure solid. ^{1}H NMR (600 MHz, $CDCl_3$) δ 3.40 (s, 1H), 2.64 (t, 2H), 2.25 (m, 2H), 2.14 (m, 2H), 1.97 (m, 2H), 1.78 (m, 2H), 1.76-1.32 (m, other aliphatic ring protons), 1.28 (s, 3H), 0.98 (s, 3H), 0.93 (s, 3H), 0.92 (s, 3H), 0.90 (s, 3H), 0.86 (s, 3H), 0.82 (s, 3H). ^{13}C NMR (150 MHz, $CDCl_3$) δ 181.57, 76.26, 60.24, 56.78, 56.68, 53.43, 50.12, 49.98, 49.03, 45.50, 42.49, 40.88, 37.53, 37.32, 36.93, 34.17, 33.28, 32.04, 29.39, 28.25, 26.97, 26.83, 25.36, 22.09, 20.77, 18.23, 18.19, 16.02, 15.91, 14.67. ESI-MS (*m/z*): 495.3 $(M+Na)^+$. Purity: 97.26%. It was analyzed by quantitative NMR and internal reference method was used.

Supplementary Scheme 2

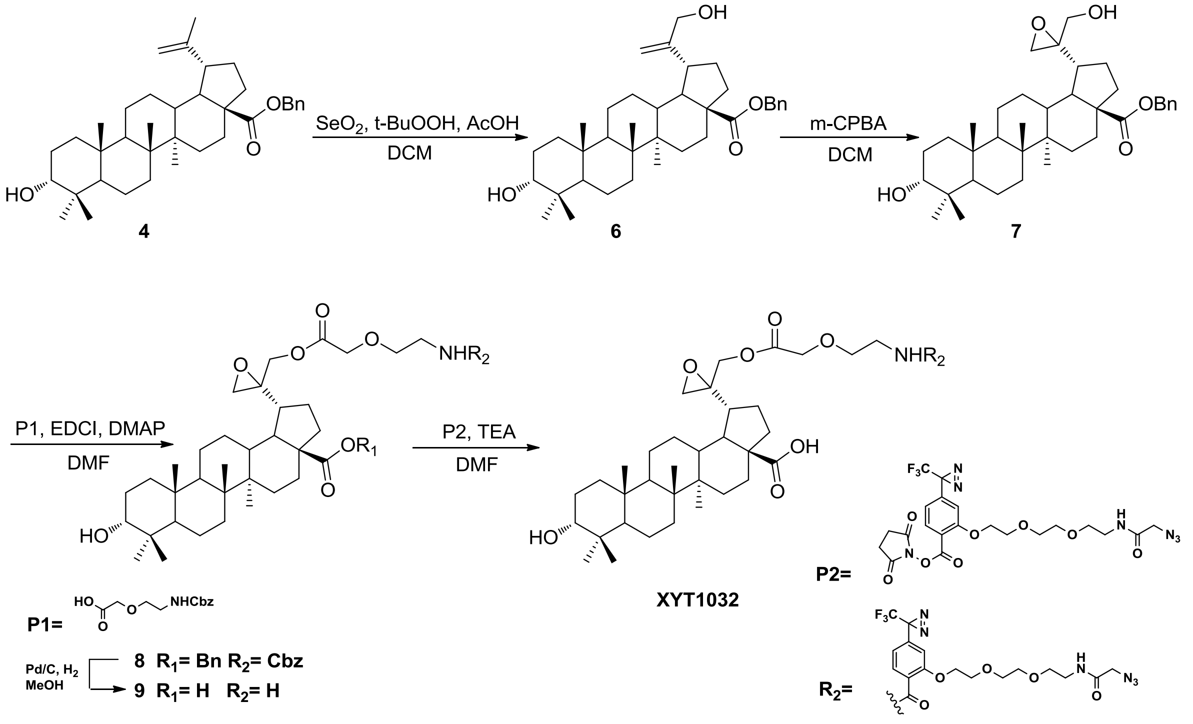

The Synthesis of photoaffinity probe XYT1032

Benzyl 3 (α)-hydroxylup-20 (29)-en-30-hydroxy-28-oate (6)

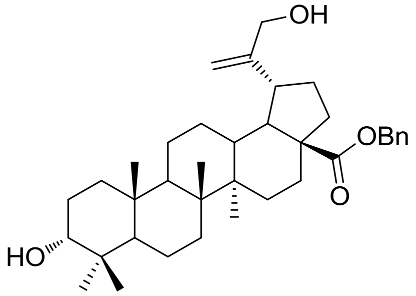

To a solution of SeO_2 (9.7 mg,0.09 mmol) and TBHP (70% solution in water, 68 μL, 0.36 mmol) in dry DCM at 0 ℃ was added AcOH(3.2 μL, 0.018 mmol) followed by dropwise addition of a solution of **4** (100 mg,0.18 mmol) in dry DCM. The reaction mixture was stirred for 12 h at ambient temperature. The reaction mixture was concentrated, extracted by EtOAc. The combined organic layers were washed with saturated Na_2SO_3 solution (2×10 mL), saturated $NaHCO_3$ solution (2×10 mL) and brine (15 mL), dried over Na_2SO_4, concentrated and purified by flash chromatography using 25% EtOAc in petroleum ether to yield 67 mg (0.12 mmol, 67%) of **6** as a white pure solid. ^{1}H NMR (300 MHz, $CDCl_3$) δ 7.34 (m, 5H), 5.10 (dd, 2H, *J* = 15.0, 12.0 Hz), 4.95 (s, 1H), 4.91 (s, 1H), 4.11 (s, 2H), 3.38 (s, 1H), 2.88 (td, 1H, *J* = 12.0, 3.0 Hz), 2.32-2.26 (m, 2H),2.21-2.10 (m, 2H), 1.98-1.77 (m, 2H), 1.64-0.96 (m, other aliphatic ring protons), 0.99 (s, 3H), 0.94 (s, 3H), 0.91 (s, 3H), 0.87 (s, 3H), 0.84 (s, 3H); ESI-MS (*m/z*): 585.4 $(M+Na)^+$.

Benzyl 3 (α)-hydroxylup-20 (29)-epoxy-30-hydroxy-28-oate (7)

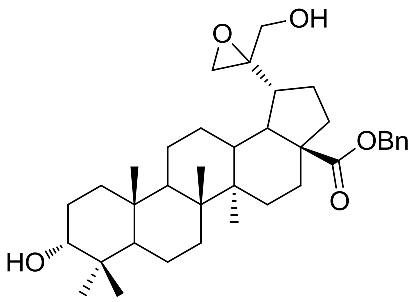

To a solution of compound **6** (200 mg, 0.36 mmol) was added m-CPBA (123 mg, 0.72 mmol) in DCM (10 mL) at ambient temperature under nitrogen atmosphere. After 3 hours, saturated Na_2SO_3 solution was added to quench the reaction, The mixture was extracted with DCM (2×50 mL). The combined organic layers were washed with H_2O (2×50 mL), saturated $NaHCO_3$ solution (2×50 mL) and brine (50 mL), dried over Na_2SO_4, concentrated and purified by flash chromatography using 33% EtOAc in petroleum ether to yield 187mg (0.32 mmol, 90%) of **7** as a white pure solid and **7** is a mixture of two diastereoisomers. ^{1}H NMR (300 MHz, $CDCl_3$) δ 7.34 (m, 5H), 5.10 (s, 2H) 3.78-3.65 (m, 2H), 3.40 (s, 1H), 2.98 (d, 1H, *J* = 6.0 Hz), 2.64 (d, 1H, *J* = 6.0 Hz), 2.35-2.24 (m, 2H), 2.01-1.92 (m, 4H), 1.86-1.75 (m, 4H), 1.57-1.28 (m, other aliphatic ring protons), 0.97 (s, 3H), 0.93 (s, 3H), 0.92 (s, 3H), 0.86 (s, 3H), 0.82 (s,3H). ESI-MS (*m/z*): 601.4 $(M+Na)^+$.

Benzyl 3 (α)-hydroxylup-20 (29)-epoxy-30-oxycarbonyl-28-oate (8)

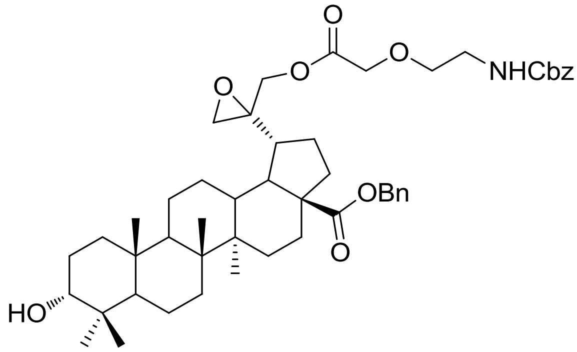

A mixture of compound **7** (31 mg, 0.054 mmol), **P1** (27 mg, 0.107 mmol) EDCI (20 mg, 0.107 mmol) and DMAP (7 mg, 0.054 mmol) in DMF (1 mL) was stirred at ambient temperature under nitrogen atmosphere for 12 h. The mixute was concentrated, diluted with H_2O (20 mL) and extracted with EtOAc (2×10 mL). The combined organic layers were washed with H_2O (2×20 mL), brine (20 mL), and dried over Na_2SO_4 concentrated and purified by flash chromatography using 33% EtOAc in petroleum ether to yield **8** (3 3mg, 0.041 mmol, 77%) as a 4:1 mixture of diastereomers in C-17. ^{1}H NMR (300 MHz, $CDCl_3$) δ 7.35-7.26 (m, 10H), 5.45 (brs, 1H), 5.10 (m, 4H) , 4.43 (d, 1H, *J* = 12.0Hz), 4.20 (s, 1H), 4.13 (s, 2H), 3.61 (t, 2H, *J* = 5.1Hz), 3.41 (t, 2H, *J* = 5.1 Hz), 3.39 (s, 1H), 2.63 (dd, 2H, J = 8.7, 4.8 Hz), 2.30 (m, 2H), 2.10 (m, 2H), 1.90 (m, 4H), 1.82 (m, 4H), 1.76-1.32 (m, other aliphatic ring protons), 0.90 (s, 3H), 0.87 (s, 3H), 0.84 (s, 3H), 0.81 (s, 3H), 0.73 (s, 3H).

Photoaffinity probe XYT1032

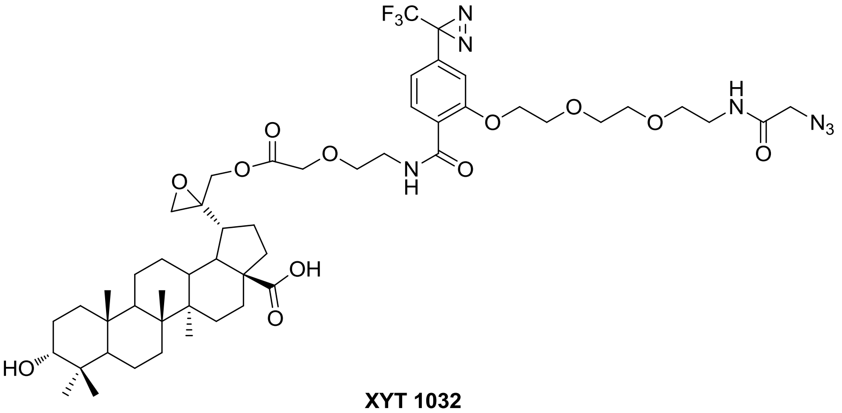

XYT 1032

To a stirred solution of **8** (50 mg, 0.061 mmol) in MeOH (1 mL), Pd/C (5 mg) was added at ambient temperature under nitrogen atmosphere. The nitrogen atmosphere was replaced by an H_2 atmosphere. The reaction mixture was stirred for 1 h at room temperature, then N_2 was replaced, and it was filtered through Celite and washed with DCM. The residue was concentrated to yield crude product **9** 30 mg (0.051 mmol, 84%) without further purification. A mixture of compound **9** (30 mg, 0.05 mmol), **P2** (27 mg, 0.05 mmol), EDCI (20 mg, 0.107 mmol) and DMAP (7 mg, 0.054 mmol) in DMF (1 mL) was stirred at ambient temperature under nitrogen atmosphere for 12 h. The mixute was concentrated, diluted with H_2O (20 mL) and extracted with EtOAc (2×10 mL). The combined organic layers were washed with H_2O (2×20 mL), brine (20 mL), and dried over Na_2SO_4, concentrated and purified by flash chromatography using DCM:MeOH=10:1 to yield **XYT1032** 18 mg (0.017 mmol, 43%). ^{1}H NMR (300 MHz, $CDCl_3$) δ 8.35 (t, 1H, *J* = 5.1 Hz), 8.18 (d, 1H, *J* = 8.4 Hz), 6.89 (d, 1H, *J* = 8.7 Hz), 6.72 (s, 1H), 4.47 (d, 1H, *J* = 12.0 Hz), 4.26 (m, 2H), 4.15 (m, 2H), 4.04 (d, 1H, *J* = 12.0 Hz), 3.95 (s, 2H), 3.93 (m, 2H), 3.71 (m, 2H), 3.66 (m, 2H), 3.57 (m, 2H), 3.50 (m, 2H), 3.40 (s, 1H), 2.82 (d, 0.2H, *J*= 6.0 Hz), 2.76 (d, 0.2H, *J* = 6.0 Hz), 2.69 (d, 0.8H, *J* = 6.0 Hz), 2.63(d, 0.8H, *J* =6.0 Hz), 2.38 (m, 2H), 2.3 3(m, 2H), 2.16 (m, 2H), 1.97-1.90 (m, 4H), 1.76-1.32 (m, other aliphatic ring protons), 0.98 (s, 3H), 0.93 (s, 3H), 0.92 (s, 3H), 0.90 (s, 3H), 0.86 (s, 3H).
